# SerpinB2 inhibits migration and promotes a resolution phase signature in large peritoneal macrophages

**DOI:** 10.1038/s41598-019-48741-w

**Published:** 2019-08-27

**Authors:** Wayne A. Schroder, Thiago D. Hirata, Thuy T. Le, Joy Gardner, Glen M. Boyle, Jonathan Ellis, Eri Nakayama, Dilan Pathirana, Helder I. Nakaya, Andreas Suhrbier

**Affiliations:** 1 QIMR Berghofer Medical Research Institute, Brisbane, Qld 4029 Australia; 2 School of Pharmaceutical Sciences, University of Sao Paulo, Sao Paulo, Brazil; 3 Department of Virology I, National Institute of Infectious Diseases, Tokyo, 162-8640 Japan

**Keywords:** Immunology, Medical research

## Abstract

SerpinB2 (plasminogen activator inhibitor type 2) has been called the “undecided serpin” with no clear consensus on its physiological role, although it is well described as an inhibitor of urokinase plasminogen activator (uPA). In macrophages, pro-inflammatory stimuli usually induce SerpinB2; however, expression is constitutive in Gata6+ large peritoneal macrophages (LPM). Interrogation of expression data from human macrophages treated with a range of stimuli using a new bioinformatics tool, CEMiTool, suggested that SerpinB2 is most tightly co- and counter-regulated with genes associated with cell movement. Using LPM from SerpinB2^−/−^ and SerpinB2^R380A^ (active site mutant) mice, we show that migration on Matrigel was faster than for their wild-type controls. Confocal microscopy illustrated that SerpinB2 and F-actin staining overlapped in focal adhesions and lamellipodia. Genes associated with migration and extracellular matrix interactions were also identified by RNA-Seq analysis of migrating RPM from wild-type and SerpinB2^R380A^ mice. Subsequent gene set enrichment analyses (GSEA) suggested SerpinB2 counter-regulates many Gata6-regulated genes associated with migration. These data argue that the role of SerpinB2 in macrophages is inhibition of uPA-mediated plasmin generation during cell migration. GSEA also suggested that SerpinB2 expression (likely via ensuing modulation of uPA-receptor/integrin signaling) promotes the adoption of a resolution phase signature.

## Introduction

SerpinB2 (aka plasminogen activator inhibitor type 2 or PAI-2) is a member of the clade B or ovalbumin-like serine protease inhibitor (ov-serpin) subgroup of the serpin superfamily. SerpinB2 is typically described as an inhibitor of the protease urokinase plasminogen activator (uPA) and (to a lesser extent) tissue plasminogen activator (tPA)^1–6^. uPA and tPA convert plasminogen to the active protease, plasmin, which has a wide range of physiological and pathological activities^7–9^. For instance, plasmin is well known for its involvement in fibrinolysis and thrombolysis, with both plasminogen activator inhibitor type 1 (PAI-1) (also known as SerpinE1) and SerpinB2 traditionally regarded as key inhibitors in these processes^3^. However, although PAI-1^−/−^ mice have clear fibrinolysis/thrombolysis defects^10–12^, evidence that SerpinB2 inhibits fibrinolysis/thrombolysis *in vivo* is actually quite limited^12^ and not without controversy^13^.

Despite over 1100 publications on PAI-2/SerpinB2, no clear consensus on the physiological function of SerpinB2 has emerged, leading to labels such as “the undecided Serpin”^14^ or the “enigmatic serpin”^4,13^. Like other ov-serpins, SerpinB2 lacks a classical secretory signal peptide and is usually found in the cytoplasm^15^, with its presumed primary target, uPA, generally localized outside the cell. How SerpinB2 might reach the extracellular milieu has been controversial^16,17^; however, SerpinB2 externalization via microparticle formation has recently been illustrated for macrophages, cancer cells and syncytiotrophoblasts^18,19^. Nevertheless, a bewildering array of intracellular and extracellular binding partners and a diversity of functions and activities have been attributed to SerpinB2^20^. For example, SerpinB2 has been reported (i) to modulate Th1/Th2 immunity^20–25^, (ii) to inhibit apoptosis in certain settings^20,26–28^, (iii) to inhibit cancer metastasis and migration^18,29–31^, (iv) to regulate differentiation and proliferation^32–36^ and (v) to inhibit IL-1β processing^37^. Although a range of uPA-independent functions for SerpinB2 have been reported^37–42^, uPA inhibition has been implicated in the majority of SerpinB2’s activities^3,12,18,31,43–45^.

SerpinB2 can be expressed by a number of different cell types including monocyte/macrophages, where SerpinB2 expression is usually inducible under inflammatory conditions^3,18,20,46^ and can reach up to 0.27% of total cellular protein^47^. However, the wide plasticity of macrophage differentiation is now well recognized, with a range of specialized macrophage populations identified in specific tissues^48–50^. Amongst these populations, constitutive expression of high levels of SerpinB2 mRNA appears to be a unique feature of large peritoneal macrophages (LPM)^51–53^. Resident peritoneal macrophages (RPM) are known to express readily detectable levels of SerpinB2 protein constitutively^25^, with RPM comprising of two subpopulations, LPM that are CD11b^hi^ and F4/80^hi^^52^ and small peritoneal macrophages (SPM) that are F4/80^lo^ and express slightly lower levels of CD11b^52,53^. Gata6 is believed to be a key transcription factor for differentiation of LPM, with two studies^52,53^ (but not a third^54^) suggesting that Gata6 induces SerpinB2 expression. An important function for F4/80^hi^, Gata6^+^ LPM was recently identified; rapid non-vascular migration from the peritoneum into sites of tissue injury, involving migration via lamellipodia extensions and ultimately the adoption of an alternatively activated phenotype to promote tissue repair^55^.

Herein we use a new bioinformatic tool, CEMiTool^56^, to identify genes in human macrophages that are usually co- and counter-regulated with respect to SerpinB2 to gain insights into SerpinB2’s function. RPM/LPM from both SerpinB2^−/−^ mice (conventional knockout) and new SerpinB2^R380A^ mice (active site mutant generated using CRISPR technology) and their respective controls were then used to further characterize the role of SerpinB2 in migration and differentiation using IncuCyte live cell analysis, RNA-Seq and gene set enrichment analyses (GSEA).

## Results

### Insights into serpinB2 function from CEMiTool analysis

**Figure 1:**
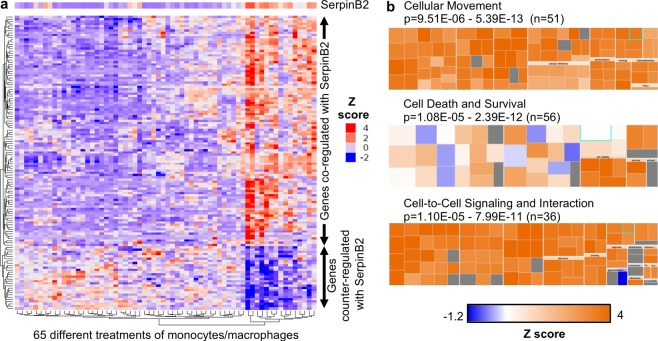
CEMiTool and IPA analyses of mRNA microarray data. (**a**) Microarray data sets from human monocytes/macrophages cultured under 65 different conditions were analyzed using CEMiTool. CEMiTool identified 129 genes (Table S1a) that were either (i) co-regulated with SerpinB2 (i.e. genes usually up-regulated when SerpinB2 was up-regulated) or (ii) counter-regulated with SerpinB2 (i.e. genes usually down-regulated when SerpinB2 was up-regulated). Expression z scores for SerpinB2 and the 129 genes after the 65 different treatments are shown. (**b**) Co-regulated genes were given a nominal expression value of 2 and counter-regulated genes were give a nominal expression value of −2, and the gene list analyzed using IPA (Direct only); 117 of the 129 genes were recognized by IPA. IPA identified *Cellular Movement* as the top scoring *Molecular and Cellular Function*; 51/117 genes appeared in 1 or more of the 96 *Categories*/*Diseases or Functions Annotations* that comprise *Cellular Movement* (Table S1b). The box plots are provided by IPA for the indicated function; each of the many (e.g. 96) internal boxes represents one annotation, with the box size indicating log_10_ p value and the color indicating the z-score.

To obtain an insight into the physiological function of SerpinB2 in human macrophages, the recently released CEMiTool^56^ was used to interrogate publicly available microarray data (GSE46903) on human peripheral blood-derived monocytes and macrophages that were stimulated *in vitro* with a range of agents, alone or in combination (n = 65 different treatments)^49^. The CEMiTool analysis identified 129 transcripts (Table S1a) that (across the 65 different treatments) were either (i) co-regulated with SerpinB2 (i.e. usually up-regulated when SerpinB2 was up-regulated) or (ii) counter-regulated with SerpinB2 (i.e. usually down-regulated when SerpinB2 was up-regulated) (Fig. 1a).

We undertook two processes to validate this CEMiTool analysis for mouse RPM. IL-6 (an important product of macrophages^50^ and important for macrophage differentiation^57^) was identified by CEMITool as usually being co-regulated with SerpinB2 (Table S1a). In agreement with this result, RPM from SerpinB2^+/+^ mice showed significantly higher IL-6 secretion than RPM from SerpinB2^−/−^ mice (Fig. S1a). Secondly, the 129 transcripts identified by CEMiTool were uploaded into Ingenuity Pathway Analysis (IPA), with 117 of these identified as genes by IPA. Co-regulated genes were given a nominal fold change of 2 and counter-regulated genes were given a nominal fold change of −2. Prominent in the top Up-Stream Regulators (USRs) identified by IPA where USRs associated with NF-κb (Fig. S1b). Consistent with these findings, significantly higher levels of DNA binding by NF-κb family members was seen in nuclear extracts from SerpinB2^+/+^ RPM than in nuclear extracts from SerpinB2^−/−^ RPM (Fig. S1c). Overall these NF-κb results are consistent with the previously published up-regulation of SerpinB2 by NF-кB in human^58^ and mouse macrophages^26^. Also identified by the IPA USR (Fig. S1b) was (i) HMGB1, the prototypical endogenous danger molecule^59^, with a positive z-score, consistent with up-regulation of SerpinB2 by pathogens and endogenous danger signals^20,60^ and (ii) the glucocorticoid receptor (NR3C1) with a negative z-score, consistent with down-regulation of SerpinB2 by glucocorticoids^61^.

The top *Regulator Effect Network* identified by IPA analysis of the CEMiTool genes was “RELA Recruitment of cells” (Fig. S1d). The top scoring *Molecular and Cellular Function* returned by IPA was *Cellular Movement* (Fig. 1b). *Cellular Movement* was returned as the top scoring *Molecular and Cellular Function* irrespective of the IPA settings (i.e. direct only or direct and indirect, with or without nominal fold change). *Cellular Movement* comprised 96 *Diseases or Functions Annotations* (Table S1b), providing the p-value range (Fig. 1b). Of the 117 genes identified by CEMiTool, a series of co-regulated chemokines (Table S1a) played a dominant role in IPA’s *Diseases or Functions Annotations* (Table S1b, Molecules), with chemokines generally associated with promotion of leucocyte migration. *Cell Death and Survival* was ranked second and *Cell-to-Cell Signaling and Interaction* third (within *Molecular and Cellular Functions)* in this analysis (Fig. 1b).

CEMiTool^56^ thus provided a unique insight into the function of SerpinB2 in human macrophages by identifying genes that were consistently co- and counter-regulated with SerpinB2 under a large range of conditions. Subsequent IPA analysis of these genes indicated that they are primarily involved in cell movement/migration, suggesting SerpinB2 is part of a transcriptional program that regulates macrophage migration.

### Peritoneal macrophage populations in serpinB2^−/−^ and serpinB2^+/+^ mice

**Figure 2:**
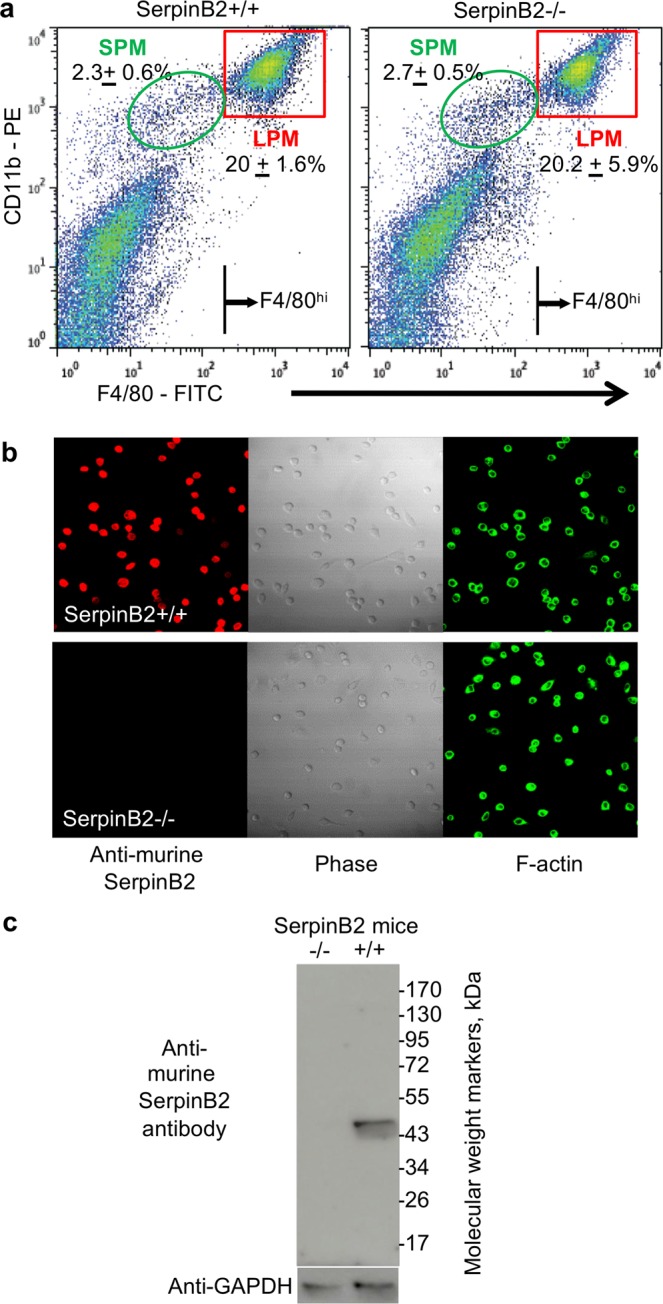
LPM in SerpinB2^−/−^ and SerpinB2^+/+^ mice. (**a**) Peritoneal lavage cells from SerpinB2^−/−^ and SerpinB2^+/+^ mice were stained with CD11b and F4/80 and analyzed by FACS. Numbers (±SD) show the percentage of SPM and LPM (gates indicated) as a proportion of all peritoneal lavage cells (n = 3 mice per group). The F4/80^hi^ lines indicate the cut-off used to FACS sort LPM. (**b**) Immunofluorescent antibody staining of FACS-sorted LPM using anti-murine SerpinB2 antibody and FITC-phalloidin (F-actin) imaged using confocal microscopy. All three images for SerpinB2^+/+^ LPM and SerpinB2^−/−^ LPM are of the same field. (**c**) Immunoblotting of LPM from SerpinB2^+/+^ and SerpinB2^−/−^ mice using anti-murine SerpinB2 and anti-GAPDH antibodies. Full length gels are shown in Fig. S15.

SerpinB2 protein is constitutively expressed by RPM, making them ideal for analyzing the function of SerpinB2 without the requirement to up-regulate SerpinB2 expression with pro-inflammatory agents^20,25^. RPM can be readily isolated from peritoneal lavage cells by adherence (see Material and Methods). Peritoneal lavage cells from C57BL/6 mice contain a large population of CD11b^hi^ and F4/80^hi^ LPM and a smaller population of CD11b^med^ and F4/80^low^ SPM cells^62^. Peritoneal lavage cells from SerpinB2^−/−^ and SerpinB2^+/+^ mice show similar proportions of LPM and SPM (Fig. 2a, red and green gates, respectively). This result was confirmed for LPM over 7 independent experiments (Fig. S2a). The number of LPM (from peritoneal lavage cells) per mouse from SerpinB2^−/−^ and SerpinB2^+/+^ mice was also not significantly different over 4 independent experiments (Fig. S2b). RPM populations from both SerpinB2^−/−^ and SerpinB2^+/+^ mice comprised ≈80% F4/80^hi^ cells (corresponding to LPM), as determined in 3–4 independent experiments (Fig. S2c). Following the convention on macrophage nomenclature^63^, we make the distinction hereafter between RPM (isolated by adherence) and LPM (identified/isolated by FACs sorting), with RPM comprising ≈80% LPM.

### LPM express serpinB2 protein

SerpinB2 protein was readily detectable in nearly all FACS-sorted LPM by confocal microscopy (Fig. 2b) using a polyclonal anti-murine SerpinB2 antibody raised against a CD interhelical loop region of murine SerpinB2^43^. No SerpinB2 protein was detected in SerpinB2^−/−^ LPM (Fig. 2b). Immunoblotting further illustrated the high level of specificity for SerpinB2 of this antibody, recognizing the expected 47 kD band in SerpinB2^+/+^ LPM lysates and showing no detectable reactivity against any protein in lysates of SerpinB2^−/−^ LPM (Fig. 2c). These data clearly illustrate SerpinB2 protein expression in LPM, consistent with constitutive SerpinB2 mRNA expression in these cells^52^. In contrast, bone marrow derived macrophages express SerpinB2 mRNA, but little if any SerpinB2 protein^25^.

FACS sorted-SPM were found not to express detectable levels of SerpinB2 protein, consistent with low levels of SerpinB2 mRNA expressed by these cells (data posted on Immgen by Gautier *et al*.)^64^. (SPM from both SerpinB2^−/−^ and SerpinB2^+/+^ mice are thus SerpinB2 negative and any differences between RPM from SerpinB2^−/−^ and SerpinB2^+/+^ mice thus likely resides within the LPM populations).

### Increased migration of serpinB2^−/−^ RPM on matrigel

**Figure 3:**
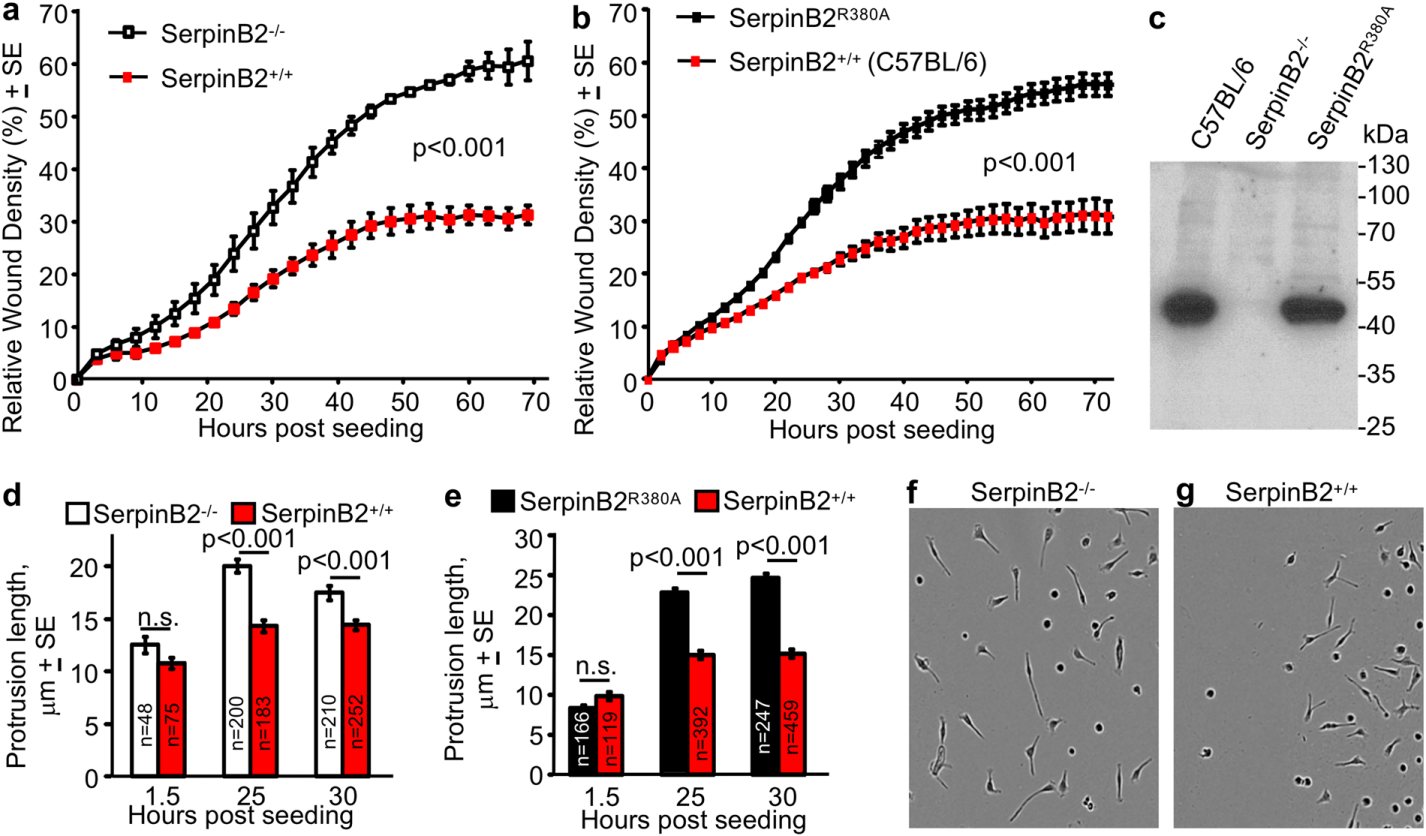
RPM migration and length of membrane protrusions. (**a**) Migration into “scratch wounds” of *ex vivo* SerpinB2^−/−^ and SerpinB2^+/+^ RPM seeded onto Matrigel-coated wells analyzed using the standard IncuCyte scratch wound assay. Data was generated (for both SerpinB2^−/−^ and SerpinB2^+/+^ RPM) from 3 pools of RPM, each derived from 3 mice, with each pool seeded into 12 wells (i.e. n = 9 SerpinB2^−/−^ and n = 9 SerpinB2^+/+^ mice). The mean of the 3 pools is shown (n = 3). Statistics by repeat measures ANOVA. (**b**) As for (**a**) but using *ex vivo* RPM from SerpinB2^R380A^ and C57BL/6J mice, one pool of 3 mice (for each strain) and 23 Matrigel coated wells. (**c**) Western blot of RPM from the indicated mice strains, stained with the murine anti-SerpinB2 antibody. (**d**) Length of cellular protrusions of RPM within the scratch areas for mice described in (**a**). The number of cells interrogated is provided within each bar (**n**), with the longest protrusion from each cell used (i.e. one value for each cell). (**e**) As for (**d**) for mice described in (**b**). (**f**,**g**) Representative phase images of the RPM described in (**d**) at 25 hours post seeding. See also Fig. S3d,e.

Given the CEMiTool results and the reported role of SerpinB2 in suppressing cancer cell metastasis^18,65^, the migration of RPM from SerpinB2^−/−^ and SerpinB2^+/+^ mice was compared using the standard IncuCyte™ scratch wound cell migration assays. RPM from SerpinB2^−/−^ mice migrated significantly faster than RPM from SerpinB2^+/+^ mice, when cells were plated onto Matrigel (Fig. 3a; a repeat experiment is shown in Fig. S3a). This effect (Fig. 3a) was not due to any cell viability differences, with SerpinB2^−/−^ and SerpinB2^+/+^ RPM after seeding onto Matrigel showing similar survival over the period of the experiment (Fig. S3b).

uPA plays a key role in migration of monocytes and macrophages via plasmin-mediated degradation of the extracellular matrix (ECM)^66,67^. These data thus support the view that SerpinB2 inhibits uPA-dependent cell migration in macrophages, with SerpinB2 externalized via microparticle formation during migration^68^ and thereby able to inhibit uPA^18^. We (like others^20^) have been unable to detect SerpinB2/uPA complexes in *in vivo* or in *ex vivo* settings. Nevertheless, RPM can be shown to secrete proteolytically active uPA, with such secretion unaffected by SerpinB2 deficiency (Fig. S3c).

### Increased migration of serpinB2^R380A^ RPM on matrigel

SerpinB2^−/−^ mice may potentially not be ideal for studying SerpinB2 deficiency for a number of reasons. The SerpinB10 gene neighbors the SerpinB2 gene on mouse chromosome 1, and a role for this gene in food allergy was recently reported^69^. C57BL/6 mice have a premature stop codon in SerpinB10^70^, whereas the original SerpinB2^−/−^ mice^71^ do not. It is highly unlikely that backcrossing onto C57BL/6^25^ would separate these two adjacent Serpin genes, so SerpinB2^+/+^ littermate mice are SerpinB10^−/−^, and SerpinB2^−/−^ mice are SerpinB10^+/+^. In addition, neighboring gene perturbation affects have previously been identified in Serpin knock-out mice^72^, with such affects potentially changing expression of other genes in the Serpin cluster. To address these potential issues, a homozygous SerpinB2 active-site mutant mouse line (SerpinB2^R380A^) on a C57BL/6 background was generated using CRISPR technology^45^, with C57BL/6 mice used as controls. As SerpinB2 has been reported to have functions that are independent of its protease inhibition activity^20,39,40,73^, the SerpinB2^R380A^ mouse would also assess the role of SerpinB2’s protease inhibition activity.

RPM from SerpinB2^R380A^ mice migrated significantly faster than RPM from C57BL/6J mice in the scratch test (Fig. 3b), clearly confirming that SerpinB2 inhibits migration on Matrigel and that SerpinB2’s protease inhibition activity is required. Immunoblotting with anti-SerpinB2 antibody showed good expression of SerpinB2^R380A^ protein in RPM from SerpinB2^R380A^ mice (Fig. 3c), illustrating that this mutation does not compromise protein expression or stability. To our knowledge, this is the first time a phenotype for a SerpinB2 reactive site mutant has been shown in primary cells. The data is consistent with uPA being the target of macrophage SerpinB2, with uPA and plasmin described as key players in macrophage migration^67,74–77^.

### SerpinB2 reduced the length of membrane protrusions in RPM

SerpinB2 expression in cancer cells has been shown to reduced migration and metastasis^18,65^ and to reduce the length of migration-associated invadopodia-like structures^18,29^. Macrophages also generate migration-associated membrane protrusions, primarily filopodia and lamellipodia. RPM membrane protrusions were measured after plating onto Matrigel and were significantly longer in SerpinB2^−/−^ RPM (Fig. 3d) and SerpinB2^R380A^ RPM (Fig. 3e) than in their respective wild-type controls. Phase images further illustrate the differences (Fig. 3f,g); additional phase images are provided in Figs. S3d,e. These data argue that SerpinB2 has a similar role in inhibiting migration in both cancer cells and macrophages.

SerpinB2 can be externalized in cancer cells and macrophages via formation of microparticles^18^. To provide evidence that extracellular SerpinB2 can indeed result in shortening of cellular protrusions, small quantities of recombinant SerpinB2 and SerpinB2^R380A^ (generated using transfected HEK239T cells) were incubated with SerpinB2^−/−^ RPM and B16 melanoma seeded onto Matrigel. Recombinant SerpinB2, but not recombinant SerpinB2^R380A^ was able (i) to form the expected covalent complex with uPA and (ii) to reduce the length of cellular protrusions in SerpinB2^−/−^ RPM and B16 cells (Fig. S4). These data support the view that extracellular SerpinB2 interacting with extracellular uPA leads to shortening of migration-associated protrusions and inhibition of migration.

### SerpinB2 localizes to focal adhesions and lamellipodia

**Figure 4:**
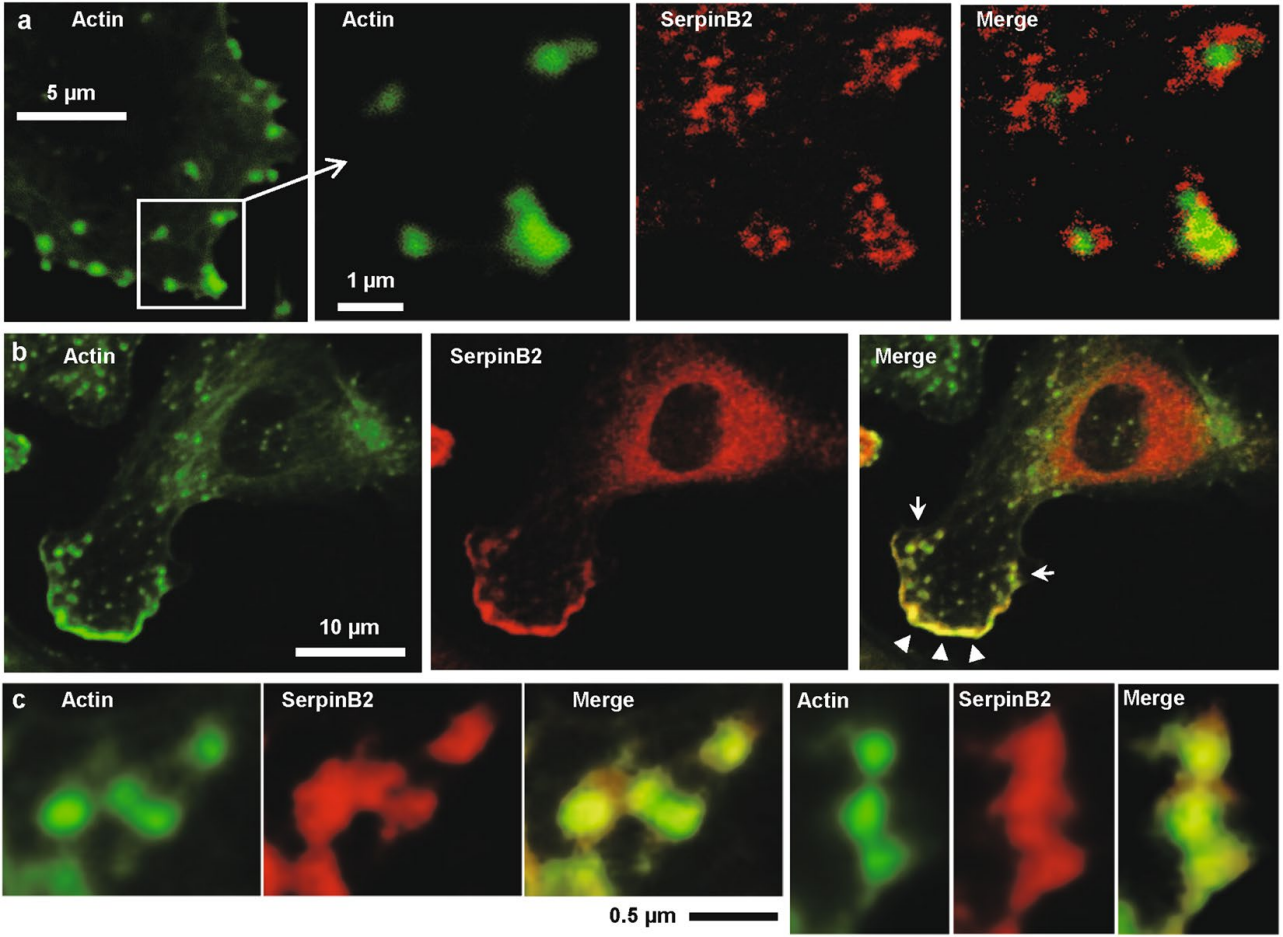
Confocal immunofluorescence microscopy. Adherent LPM from wild-type mice were dual labeled with FITC-phalloidin and anti-SerpinB2 antibody. (**a**) Actin; FITC-phalloidin (F-actin) staining showing actin concentrations at focal adhesions (green). SerpinB2; SerpinB2 staining localized around and in the focal adhesions (red). Merge; overlapping staining (yellow); see Fig. S5 for enlargements and quantitation of overlap. (**b**) Actin; FITC-phalloidin staining showing F-actin concentration along the leading edge of a lamellipodia (bottom left, green). SerpinB2; SerpinB2 staining (red) also staining the leading edge of a lamellipodia (bottom left, red). Merge; overlapping staining at the leading edge of a lamellipodia (yellow, arrowheads). (**c**) The two clusters of focal adhesions indicated in b (arrows) are enlarged to show overlapping localization of actin and SerpinB2 staining. The images shown are representative of 2 independent experiments where 5 images were examined containing ≈10 cells per image with ≈50 cells examined per experiment. Approximately 30% of cells examined showed overlapping staining at focal adhesions and/or lamellipodia.

Microparticle formation has been reported to be intimately associated with formation of membrane protrusions during migration^68^. Using confocal microscopy of SerpinB2^+/+^ RPM, SerpinB2 was consistently found adjacent to, or close to, actin in focal adhesions and lamellipodia (Fig. 4). Enlargements of the four focal adhesions in the merged image in Fig. 4a (right hand image) are shown in Fig. S5. Quantitation of green (actin), red (SerpinB2) and yellow (merged) pixels are provided, with yellow pixels comprising about 10–25% of all green/red/yellow pixels (Fig. S5). The close proximity of actin and SerpinB2 could also be in seen in the leading edge of lamellipodia (Fig. 4b, Merged, white arrowheads). Just behind the leading edge, the close proximity of actin and SerpinB2 could again be seen in focal adhesions (Fig. 4b, Merged, white arrows), with enlarged images of these focal adhesions again showing significant levels of overlapping staining (Fig. 4c). Both uPA, the uPA receptor and plasmin are found associated with focal adhesions and lamellipodia^66,78,79^, with plasmin generation required for degradation of the ECM and de-adhesion^67,78,80,81^. Thus SerpinB2 localizes to sites associated with microparticle formation, uPA activity and migration, consistent with the view that SerpinB2 inhibits migration by inhibiting uPA^18,65,82^.

The generalized uPA-dependent peri-cellular proteolysis reported previously for macrophages^67^, was not increased in SerpinB2^−/−^ RPM (Fig. S6). This observation is consistent with the lack of an effect of SerpinB2 expression on extracellular levels of active uPA (Fig. S3c). uPA-dependent peri-cellular proteolysis is not ordinarily associated with microparticle release, arguing that SerpinB2-mediated inhibition of uPA is restricted to settings where microparticle formation allows SerpinB2 externalization, such as during the formation and retraction of membrane protrusion that is associated with migration^68^.

### RNA-Seq of migrating RPM from SerpinB2^R380A^ and C57BL/6 mice

RPM from SerpinB2^R380A^ and C57BL/6 mice migrating on Matrigel (as in Fig. 3b) for 24 hours were analyzed by RNA-Seq. Library size graphs and MDS plots are shown in Fig. S7a,b. A complete list of gene counts are provided in Table S1c, from which a list of differentially expressed genes (DEGs) was generated (q < 0.01, n = 1481) (Table S1d). Validation by qRT PCR of differentially expression of 4 genes is shown in Fig. S7c–f.

**Figure 5:**
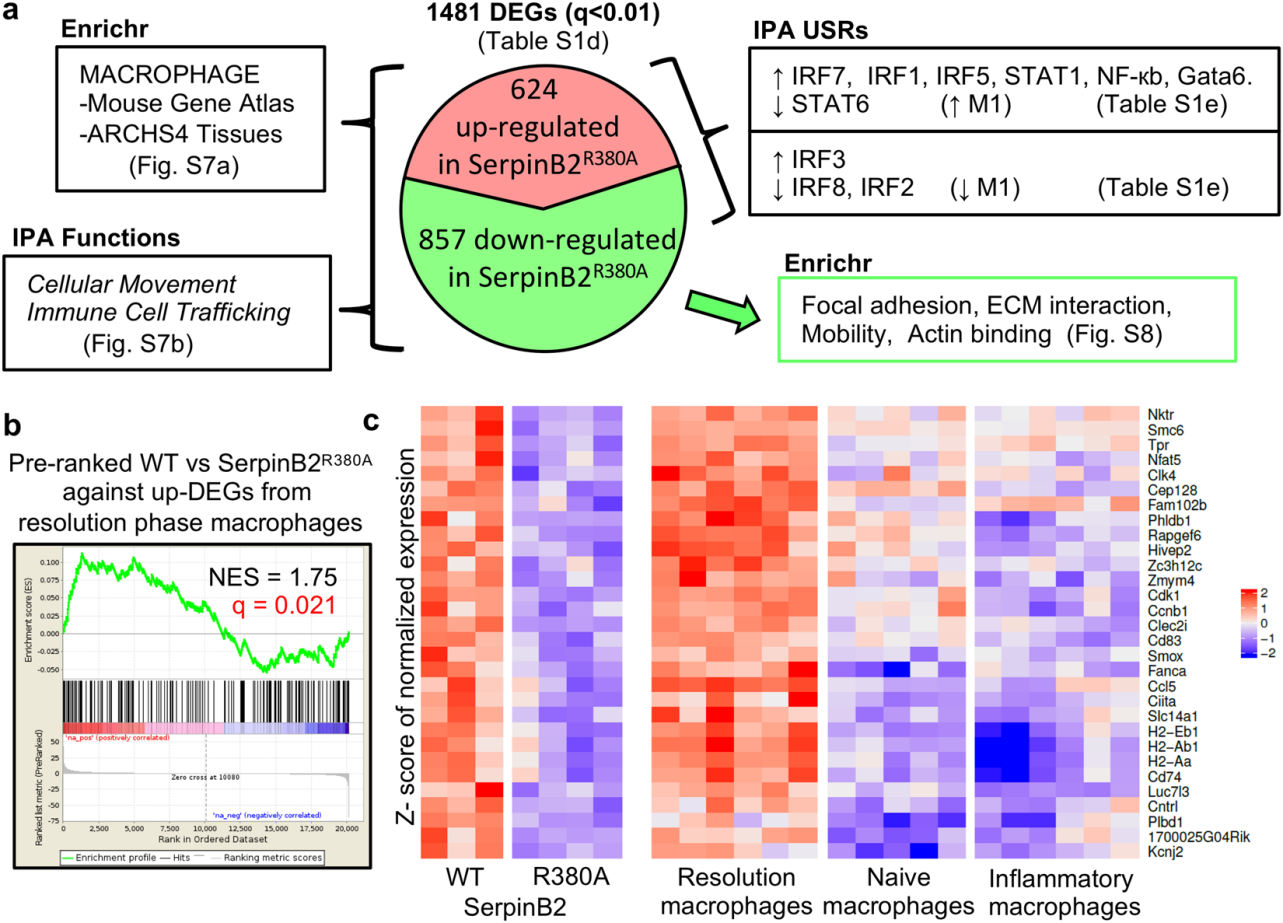
Summary of RNA-Seq data and pre-ranked GSEA for resolution phase macrophages. (**a**) RPM from SerpinB2^R380A^ and C57BL/6 mice were seeded onto Matrigel for 24 h and were then analyzed by RNA-Seq; 1481 DEGs were identified (using a filter of q < 0.01); 624 DEGs were up-regulated, and 857 were down-regulated in SerpinB2^R380A^ RPM. These DEGs were analyzed by Enrichr and IPA. (**b**) Up-regulated DEGs from resolution phase macrophages (Table S1g) were analyzed by GSEA against the complete RNA-Seq gene list (Table S1c), with genes pre-ranked by p-value. The NES score (normalized enrichment score) and false discovery rate (FDR) q values are indicated. (**c**) Heat map of core enriched genes from b, illustrating that genes up-regulated in wild-type (WT) RPM (relative to SerpinB2^R380A^ RPM) are also up-regulated in resolution phage macrophages (relative to naïve and inflammatory macrophages). The core enriched gene list is also provided in Table S1g.

The aforementioned 1481 DEGs were analyzed using Enrichr, with the Mouse Gene Atlas and ARCHS4 Tissues data bases returning *Macrophage* as the top term (Figs 5a, S8a); consistent with the FACS data showing that these RPM cultures comprise >80% F4/80^hi^ macrophages (Fig. S2c). The fibrinolysis pathway was also returned as the top term by BioCarta 2013 (Fig. S8a, blue arrow). When the DEG list was analyzed by IPA the most significant *Molecular and Cellular Function* was *Cellular Movement* and the most significant *Physiological System Development and Function* was *Immune Cell Trafficking* (Figs 5a, S8b); consistent with the results in Figs 1b and 3b.

When the DEG list (n = 1481; Table S1d) was further analyzed using Enrichr, multiple data bases returned top ranking terms associated with focal adhesions, integrin signaling and ECM interactions; consistent with Fig. 4. These terms emerged to be largely present in the down-regulated genes (n = 857) (Figs 5a, S9); an observation consistent with reduced matrix interactions for faster migrating SerpinB2^R380A^ RPM (Fig. 3b). Panther 2016 also returned Plasminogen activating cascade (Fig. S9, blue arrow).

### SerpinB2 and M1/M2 polarization

When the DEG list (n = 1481; Table S1d) was analyzed using IPA upstream regulator function (USR, direct only), a series of M1-associated USRs (see below) were identified (Fig. 5a, Table S1e) that were largely retained when only the up-regulated genes (n = 624) were used (Table S1f). Amongst the USRs was RELA (Table S1e), consistent with Fig. S1d and the increased migration seen for SerpinB2^R380A^ RPM (Fig. 3b). Particularly prominent (high activation z-scores) amongst the USRs that were more active in SerpinB2^R380A^ RPM, were a series of transcription factors associated with M1-polarization^83,84^; specifically, interferon response factors (IRF7, IRF1 and IRF5), STAT1 and NF-кb family members (REL, NFKB2, RELA, NFKB1) (Fig. 5a, Table S1e). STAT6, an inhibitor of M1 polarization^84^, was also identified with a negative activation z-score (Table S1e). These results are arguably consistent with multiple studies in SerpinB2^−/−^ mice reporting that in certain settings SerpinB2 can regulate Th1/Th2 immunity^20–25^, with Th1 associated with M1 and Th2 associated with M2 macrophage polarization^85^. However, IRF3 (generally associated with M2^84^) is also predicted to be more active in SerpinB2^R380A^ RPM, and IRF8 and IRF2 (that usually promote M1^84^) were identified with negative z-scores (Fig. 5a, Table S1e). These results suggest that SerpinB2’s effects on macrophage polarization are not a straightforward fit with the classical M1/M2 model^50^. This notion is supported by qRT-PCR analysis showing no significant differences in the mRNA levels of the classical M2 marker, Arg-1, in SerpinB2^−/−^ and SerpinB2^+/+^ RPM (Fig. S10a). Macrophage polarization has been associated with changes in phagocytic activity^86–88^ and phagocytosis of carboxylate-modified polystyrene beads^88^ was also unaffected in SerpinB2^−/−^ mice (Fig. S10b).

### SerpinB2 expression promotes adoption of a resolution phase signature

Gata6+ LPM ultimately adopt an alternatively activated phenotype as part of the tissue repair process^55^. To test whether loss of SerpinB2 function might influence this process, a list of 224 genes up-regulated in mouse resolution phase peritoneal macrophages (relative to both naive peritoneal macrophages and inflammatory peritoneal macrophages) (Table S1g) was generated from publicly available microarray data, using data and methods described in Stables *et al*.^89^. The 224 DEGs were then used in a GSEA against a pre-ranked gene list of all genes from the RNA-Seq analysis of C57BL/6 versus SerpinB2^R380A^ RPM. A significant correlation emerged (Fig. 5b), suggesting SerpinB2 expression promotes up-regulation of a number of genes associated with the resolution phase signature in peritoneal macrophages (Fig. 5c). Core enriched genes (n = 31) (Fig. 5c) analyzed by IPA (direct and indirect) returns IL-27 (p = 3.9 × 10e8, activation z-score = 2.4), IL-6 (p = 4.4 × 10e8, activation z-score = 1.5) and STAT3 (p = 1.1 × 10e6, activation z-score = 0.58) as top USRs by p value. IL-27, IL-6 and STAT3 are associated with alternative activation and/or anti-inflammatory activity^57,90^. This bioinformatics analysis is consistent with the significantly elevated levels of IL-6 secreted by cultured SerpinB2^+/+^ compared to SerpinB2^−/−^ RPM (Fig. S1a). Taken together these results argue that SerpinB2 expression in LPM contributes to the alternatively activated tissue repair program adopted by LPM at the site of injury^55^.

### SerpinB2 and Gata6

**Figure 6:**
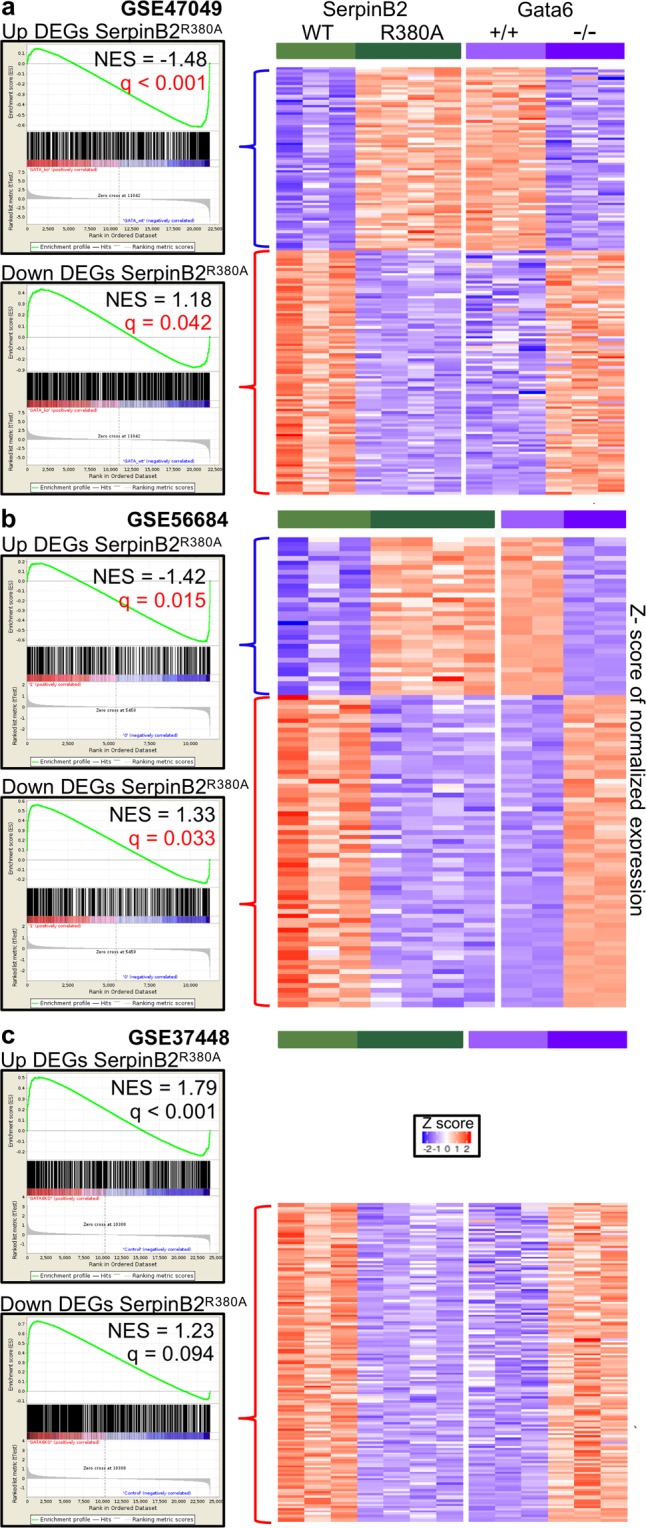
GSEA using microarray data sets from Gata6^−/−^ LPM. The 624 up-regulated and the 857 down-regulated DEGs in SerpinB2^R380A^ RPM were compared by GSEA with 3 microarray data sets that compared LPM from Gata6^+/+^ and Gata6^−/−^ mice; (**a**) GSE47049, (**b**) GSE56684 and (**c**) GSE37448. GSEAs showing significant counter-regulation (i.e. up in SerpinB2^R380A^ and down in Gata6^−/−^ or down in SerpinB2^R380A^ and up in Gata6^−/−^) are indicated with red p values. Heat maps of core enriched genes showing counter-regulation are shown for (**a**,**b**) and for Down DEGs in (**c**), which showed counter-regulation that did not reach significance (q = 0.094). Up DEGs in (**c**) showed co-regulation (positive NES score) indicating no significant counter-regulation. The core enriched genes illustrated by the heat maps are listed in Table S1h.

Two studies^52,53^ (but not a third^54^) suggested that Gata6 induces SerpinB2 expression. The USRs described above also include Gata6 (Table S1e, z score = 2.3, p = 9.8 × 10^−9^), suggesting loss of SerpinB2 activity increased the activity of Gata6. GSEAs were thus undertaken comparing DEGs from SerpinB2^R380A^ RPM (Table S1d) with the published microarray analyses from studies on Gata6^−/−^ LPM. For GSE47049^53^, up-regulated DEGs from SerpinB2^R380A^ RPM showed significant correlation with genes down-regulated in Gata6^−/−^ LPM (Fig. 6a). In addition, down-regulated DEGs from SerpinB2^R380A^ RPM showed significant correlation with genes up-regulated in Gata6^−/−^ LPM (Fig. 6a). The same patterns emerged for GSE56684^52^ (Fig. 6b). The third study GSE37448^54^, which did not identify SerpinB2 as a Gata6-regulated gene, showed significant co-regulation (rather than counter-regulation) for up-regulated genes (Fig. 6c, Up DEGs). For down-regulated genes, core enriched genes again showing clear counter-regulation (with considerable overlap with the other studies), although this did not reach significance (Fig. 6c, Down DEGs). A very similar picture merged when the same GSEAs were undertaken using DEGs (listed in Table S1i) obtained from RNA microarray analyses of *ex vivo* FACS-sorted F4/80^hi^ cells (gate is shown in Fig. 2a) from SerpinB2^−/−^ and SerpinB2^+/+^ mice (Fig. S11). Thus transcriptome analysis of migrating SerpinB2^R380A^ RPM and *ex vivo* SerpinB2^−/−^ F4/80^hi^ cells both suggest that a significant number of genes regulated by Gata6 are counter-regulated by SerpinB2 expression.

The core enriched genes from GSEAs using GSE47049 and GSE56684 (Table S1h) for DEGs up-regulated in SerpinB2^R380A^ RPM (Fig. 6a,b, Up DEGs) were analyzed by IPA (direct only). The top scoring *Molecular and Cellular Functions* was *Cellular Movement* (p-value range 2.49e-03 - 4.03e-09). When the core enriched genes (Table S1h) from all 3 studies for DEGs down-regulated in SerpinB2^R380A^ RPM (Fig. 6a–c, Down DEGs) were analyzed by IPA, the top *Physiological System Development and Function* was *Immune Cell Trafficking* (1.50e-03 - 1.02e-08). In broad terms these results are consistent with a role for Gata6 in promoting migration^91–95^ and SerpinB2-expression inhibiting migration. We speculate about a potential mechanism that might underpin this counter-regulation in Fig. S12 (see Discussion).

### SerpinB2 and RPM survival

uPA and plasmin have been linked to apoptosis in a variety of cell types^96,97^ including monocytes/macrophages^98–100^ and cancer cells^27,101,102^, with the latter studies illustrating that uPA/plasmin can both promote and suppress apoptosis depending on the setting. SerpinB2 has similarly been reported to both inhibit^20,26,41,42^ and promote apoptosis^103,104^, with several studies also showing no effect on apoptosis^18,43,105^. We were unable to see any differences in cell death of SerpinB2^−/−^ and SerpinB2^+/+^ RPM induced by LPS, IFNγ, ATP or L-Leucyl-L-Leucine methyl ester (Fig. S13). Viability on Matrigel was also unaffected by SerpinB2 deficiency (Fig. S3b).

### IL-1β induction and secretion was unaffected in SerpinB2^−/−^ RPM

SerpinB2 has been reported to inhibit caspase 1^42^ and IL-1β processing^37^. However, we were unable to see any significant increases in pro-IL-1β expression or IL-1β secretion in SerpinB2^−/−^ RPM (Fig. S14). We also previously saw no role for keratinocyte SerpinB2 in IL-1β expression or processing^43^.

## Discussion

Herein we provide multiple lines of evidence that the function of macrophage SerpinB2 is inhibition of migration. The data was derived from bioinformatics analyses and investigation of *ex vivo* murine LPM/RPM that constitutively express SerpinB2. LPM from two SerpinB2-deficient mouse strains (and their respective wild-type controls) were used, (i) conventional knock-out SerpinB2^−/−^ mice and (ii) SerpinB2^R380A^ mice generated using CRISPR technology. Macrophage SerpinB2 thus appears to have a similar function to SerpinB2 expression in cancer cells, where in a range of settings SerpinB2 expression has been shown to inhibit metastasis/invasion^18,29,31,44,65,82^.

A large body of evidence supports a role for uPA and plasmin in promoting migration^66,67,74–77,106^. That SerpinB2 can inhibit uPA *in vitro* is not in dispute^3^ (Fig. S4b). That SerpinB2 inhibits uPA *in vivo* has been less easy to demonstrate^20^, with SerpinB2-uPA complexes (to the best of our knowledge) - demonstrated in *ex vivo* samples in only one^45^ (perhaps two^107^) publications. We have also been unable unequivocally to demonstrate such complexes from primary macrophages, perhaps because most such complexes are not cell associated, are present at low concentrations and/or because they are rapidly degraded^108^. The Enrichr analyses of the RNA-Seq data did provide some evidence for a role for uPA/plasmin, with 2 data bases, BioCarta 2013 (Fig. S7A, blue arrow) and Panther 2016 (Fig. S8, blue arrow), identifying fibrinolysis/plasminogen activation, although neither reached significance after multiple comparisons adjustment of the p-value. However, the general (and perhaps inappropriate^13^) assumption that SerpinB2 (like PAI-1) is associated only with fibrinolysis, may have resulted in a paucity of appropriate data and data-base annotation. The evidence that SerpinB2 inhibits uPA *in vivo* thus remains largely indirect. The increased migration of SerpinB2^R380A^ RPM provides compelling evidence that the protease inhibition function of SerpinB2 is involved; an observation consistent with uPA inhibition. SerpinB2 and actin staining overlapped in focal adhesions and lamellipodia, structures that are also associated with uPA, the uPA receptor and plasmin^66,78,79^. SerpinB2 can be externalized via microparticle formation^18^, a process intimately associated with formation of membrane protrusions during migration^68^. In addition, rSerpinB2 (but not rSerpinB2^R380A^) formed complexes with uPA and reduced the length of cellular protrusions (Fig. S4), with SerpinB2 (but not SerpinB2^R380A^) expression similarly reducing the length of migration-associated cellular protrusions (Fig. 3d,e).

There is a widespread view that SerpinB2 inhibits apoptosis^20,26,41,42^, although this activity appears to be context dependent^18,43,103–105^. The evidence presented herein may provide an explanation, given that migration and ECM attachment can influence susceptibility to apoptosis^109,110^, with uPA and plasmin also linked to apoptosis in a variety of cell types^96,97^ including monocytes/macrophages^98–100^ and cancer cells^27,101,102^. Increased SerpinB2 may simply reduce plasmin-mediated ECM degradation and cell detachment, thereby promoting cell survival^111^. For instance, SerpinB2 may inhibit cell death by reducing signaling that promotes anoikis (ECM detachment-induced apoptosis)^112^.

We have been unable to find any evidence in keratinocytes^43^ or in RPM (Fig. S14) that SerpinB2 regulates IL-1 processing^37,113^. However, IL-1α and IL-1β were identified by CEMiTool as being consistently co-regulated with SerpinB2 (Table S1a). Both SerpinB2 and IL-1 are externalized (secreted) under pro-inflammatory conditions by non-conventional processes involving secretory vesicles or via loss of the plasma membrane barrier^18,43,114,115^, although the role of gasdermin D pores (that facilitate IL-1β secretion^114^) in SerpinB2 secretion from macrophages remains to be determined. Thus, although SerpinB2 does not appear to regulate IL-1, SerpinB2 and IL-1 often appear to be co-regulated and may be secreted by similar mechanisms.

Counter-regulation of some Gata6-regulated genes associated with cell migration by SerpinB2 expression is a novel finding, although the observation is consistent with (i) promotion of cell migration via elevated levels of Gata6 in a number of settings^91–95^, perhaps mediated in part by direct up-regulation of uPA^116,117^ and (ii) inhibition of migration by SerpinB2 via reduced uPA-mediated plasmin formation, with plasmin associated with promotion of migration^106^. The mechanistic basis of this counter-regulation remains to be characterized; however, one might speculate that ERK is involved. SerpinB2^+/+^ RPM express lower levels of phospho-ERK1/2 than RPM from SerpinB2^−/−^mice (Fig. S12a). Less phospho-ERK1/2 activity would reduce phosphorylation and activation of FOXO1^118^, with a SerpinB2-associated reduction in FOXO1 activity supported by IPA analysis of the core enriched genes from Fig. 6 (Fig. S12b). FOXO1 has recently been shown positively to regulate Gata6 expression in peritoneal resident macrophages^119^, so reduced FOXO1 would lead to reduced Gata6 expression (Fig. S12c). Less GATA6 activity in SerpinB2^+/+^ RPM is also predicted by IPA analysis of the RNA-Seq data (see Table S1e; showing more GATA6 activity in SerpinB2^R380A^ RPM).

That changes in migration might contribute to altered macrophage differentiation is perhaps not surprising^120–122^. Simple inhibition of plasmin production would appear unlikely to provide a straightforward mechanism, as plasmin has been reported to promote both M1/pro-inflammatory responses^123–125^ and M2-associated macrophage polarization^126,127^. Modulation of integrin and uPAR signaling events are more likely involved^44,128–133^. For instance, uPAR/integrin signaling often involves ERK activation^44,129,131,134^. Phospho-ERK-1/2 levels were higher in SerpinB2^−/−^ RPM (Fig. S11a), consistent with observations in cancer cells^135^. ERK activation is often associated with M1 polarization^136–139^ and migration^140,141^. SerpinB2 has been associated with suppression of some M1-associated activities in certain settings^20–25^; however, the overall fit with the M1/M2 paradigm appeared to be poor (Fig. 5a). The key observation made herein is that SerpinB2-associated modulation of macrophage differentiation perhaps has less to do with M1/M2; instead, SerpinB2 expression appears to inhibit migration and promote adoption of a resolution phase signature (Fig. 5b), consistent with the role of LPM in tissue repair^55^.

## Materials and Methods

### CEMiTool analysis

CEMiTool^56^ was used to analyze microarray data (GSE46903) on human peripheral blood-derived monocytes and macrophages stimulated *in vitro* with 65 different treatments^49^. The raw microarray gene expression data was downloaded from the Gene Expression Omnibus website (GEO, www.ncbi.nlm.nih.gov/geo/). The arrayQualityMetrics Bioconductor package^142^ was used to remove samples that failed at least 3 of the 5 tests. The expression normalization was performed by the affy package’s RMA function (Bioconductor)^143^. CEMiTool was used with default parameters to identify co-expression modules using normalized expression data^56^; specifically to identify genes that were co-regulated or counter-regulated with SerpinB2.

### IPA analyses

Data were analyzed through the use of Ingenuity Pathway Analysis (IPA)^144^ (QIAGEN Inc., https://www.qiagenbioinformatics.com/products/ingenuity- pathway-analysis). Ingenuity Pathway Analysis was undertaken using default parameters (direct only or direct and indirect, as stipulated for each analysis) using Build version 478438 M, Content version 44691306 (Release Date 2018-06-15).

### Ethics statement

Mouse work was approved by the QIMR Berghofer Medical Research Institute Animal Ethics Committee. All mouse work was conducted in accordance with the “Australian code for the care and use of animals for scientific purposes” as defined by the National Health and Medical Research Council of Australia. Mice were euthanized using carbon dioxide.

### Mice

SerpinB2^−/−^ and SerpinB2^+/+^ mice backcrossed 12 times onto C57BL/6 J mice have been described previously^25^ and were bred in-house at QIMR Berghofer. Heterozygous mice with a SerpinB2^R380A^ active site mutation were generated at the Australian Genome Research Facility Ltd. (Melbourne, Australia). The active site (P1) Arg380^20^ (codon AGA) of SerpinB2 was changed to Ala380 (codon GCA); i.e. nucleotides 1222 and 1223 (with reference to accession NM_011111.4) were changed from AG to GC. Two silent mutations were also introduced GCAACTGGACATGGTGGCCCACAGTTTGTC to prevent cutting of the oligonucleotide during genome editing. A homozygous SerpinB2^R380A^ line was generated in-house. The genotype was confirmed by tail tipping, extraction of DNA (Extract-N-Amp Tissue PCR Kit, Sigma), PCR (primers Forward tctgaggtgttccatcaag, Reverse ctaccaacaaatagtatcgtgtg) and sequencing of the PCR products^45^.

### Isolation of peritoneal lavage cells

Peritoneal lavage cells were isolated essentially as described^145^. Female mice were used to avoid any potential issues arising from fighting and injury occasionally seen in male mice. RPM were isolated by adherence as described^25^; see also Fig. S2.

### FACS analysis

FACS analysis of peritoneal lavage cells used the LSR Fortessa 4 (BD Biosciences) and FACS sorting the MOFLO XDP (Beckman Coulter, Inc.), with analysis undertaken using FLowJo vX.0.7 software. Antibodies used were APC anti-mouse CD11b (Cat 101212, clone M1-70, IgG2b, Biolegend), isotype control APC Rat IgG (Cat 400612, clone RTK4530, IgG2b, Biolegend), FITC anti-mouse F4/80 (Cat MCA497FB, clone CI:A3-1, IgG2b, AbD Serotec), and isotype control FITC Rat IgG (Cat 400505, clone RTK2758, Biolegend).

### IncuCyte scratch wound cell migration assay

Peritoneal lavage cells were seeded onto wells of 96 well plates (ImageLock, Essen Bioscience Inc, MI, USA) coated with Corning Matrigel Matrix (Corning Incorporated, Tewksbury, MA, USA) (overnight 100 μg/ml). Cells were incubated for 3 hours; medium RPMI1640 supplemented with 10% endotoxin free (tested using RAW264 reporter cells^146^) fetal calf serum (Gibco), glutamine (Gibco) and penicillin/streptomycin (Thermo Fisher Scientific). Wells were washed 3 times in PBS. The plates were analyzed at the indicate times via the IncuCyte ZOOM System (Essen Bioscience) and the 2016A live cell analysis software after application of a scratch. The Relative Wound Density (%) over time was determined with the cell density in the scratch wound area expressed relative to the cell density outside of the scratch wound area.

The lengths of cellular protrusions from RPM within the scratch wound areas were measured using the images provided by IncuCyte (and uploaded into ImageJ), with the longest protrusion from each cell used (i.e. one value for each cell). Round cells (protrusion length equal to zero) were not included as these cells were deemed to be non-migrating.

### Anti-mouse serpinB2 antibody

An affinity purified rabbit polyclonal, anti-murine SerpinB2 antibody was supplied by Peptide Specialty Labs GmbH (Heidelberg, Germany). A coupled CD loop region peptide EIGSYGITTRNPENFSGC was used as the immunogen^43^. Immunoblotting was performed as described^43^.

### Confocal florescent microscopy

Confocal microscopy was undertaken using Leica TCS confocal microscope (Leica Microsystems, North Ryde, NSW, Australia), with images shown using 2–3 adjacent 500 nm Z stacks. Staining with the anti-mouse SerpinB2 antibody and phalloidin-rhodamine (for actin) has been described previously^18^. Briefly cells were cultured on glass coverslips, fixed in 2% paraformaldehyde and permeabilized with 0.1% Triton X-100 in PBS.

### RNA-Seq

Peritoneal lavage cells from SerpinB^R380A^ and C57BL/6 mice were seeded onto Matrigel in 6 well plates; 10^7^.cells from 3 mice per well (3 wells per mouse strain as biological replicates). After 3 hours plates were extensively washed with PBS. After 24 hours culture, RPM were extracted using RNeasy Plus Mini kit (including a RNase free DNase I step) according to manufacturer’s instructions. Samples were sent to the Australian Genome Research Facility (Melbourne, Australia), cDNA libraries were prepared and sequenced (100 bp single end reads) using the Illumina HiSeq2500 Sequencer (Illumina Inc.) with reads mapped to the mouse genome (Mus_musculus.GRCm38) as described^147^. The read counts were used to determine gene expression and identify differentially expressed genes (DEGs) using R packages (R version 3.2.0) ‘edgeR’ (v3.18.1) and ‘limma’ (3.32.7). (https://bioconductor.org/packages/release/bioc/html/edgeR.html). The default TMM normalization method of edgeR was used to normalize the counts. The GLM model was used to perform differential expression comparison between the groups. Genes that had >1 CPM in at least 3 samples were used for further analysis. The differentially expressed gene list was generated by applying a Benjamini-Hochberg corrected p-value (i.e. FDR or q value) filter of <0.01.

### Gene set enrichment analysis (GSEA)

GSEAs^148^ were conducted using the software GSEA Desktop v3.0 (Broad Institute Inc., MIT, USA). A pre-ranked GSEA was performed to determine whether the up-regulated genes from resolution phase macrophages^89^ were enriched in the SerpinB2^R380A^ RPM expression dataset (Fig. 5b). Resolution phase macrophages in the Stables *et al*. publication^89^ were obtained by purification of macrophages from peritoneal lavage cells by B cell depletion and adherence >48 hours after intraperitoneal treatment with 0.1 mg zymozan. They were distinguished from both naïve macrophages (no treatment) and pro-inflammatory macrophages (intraperitoneal treatment with 10 mg zymozan). The RNA-Seq expression ranked file contained genes with >1 CPM in at least 3 samples. The gene rank values were calculated by -log10(p-value) from DEG analysis (limma package, Bioconductor), where the up-regulated genes (in WT) were given a positive p-value and down-regulated genes given a negative p-value. The resolution phase gene set (Table S1g) was generated using the microarray data (Gene Express; E-MEXP-3189) and methods described previously^89^. The parameters used in the pre-ranked GSEA were the classic enrichment statistic, tTest metric, with 1,000 gene set permutations. GSEA was also used to determine whether up or down-regulated genes in WT versus SerpinB2^R380A^ RPM were enriched in 3 expression sets from Gata6^−/−^ mice (Fig. 6), with the GSEAs using weighted p2 enrichment statistic. The raw microarray gene expression data was downloaded from the GEOwebsite and process as described above for CEMiTool.

### Statistics

Statistical analysis of experimental data was performed using IBM SPSS Statistics for Windows, Version 19.0. The t test was used when the difference in variances was <4, skewness was ≥2 and kurtosis was ≤2. When differences in variances were >4 the non-parametric Kolmogorov–Smirnov test was used. The repeat measure ANOVA was used for IncuCyte data (Fig. 3a,b) given cells were repeatedly imaged over time.

## Supplementary information

Supplementary Figures

Table S1

## Data Availability

The Illumina RNA-Seq data generated for this study are available from NCBI SRA accession: SRP158653.
