# Supplementary material for: SerpinB2 inhibits migration and promotes a resolution phase signature in large peritoneal macrophages
Source: Sci Rep. 2019 Aug 27;9:12421. doi: 10.1038/s41598-019-48741-w (PMC6712035; doi:10.1038/s41598-019-48741-w)
Supplement: Supplementary file 1 — Supplementary Figures [file 41598_2019_48741_MOESM1_ESM.pdf]

SerpinB2 inhibits migration and promotes a resolution phase signature in large peritoneal macrophages

Wayne A Schroder[1#], Thiago D Hirata[2#], Thuy T Le[1#], Joy Gardner[1], Glen M Boyle[1], Jonathan Ellis[1], Eri Nakayama[3], Dilan Pathirana[1], Helder I Nakaya[2] and Andreas Suhrbier.[1*]

[1] QIMR Berghofer Medical Research Institute, Brisbane, Qld. 4029, Australia.
[2] School of Pharmaceutical Sciences, University of Sao Paulo, Sao Paulo, Brazil.
[3] Department of Virology I, National Institute of Infectious Diseases, Tokyo 162-8640, Japan.

[*]Corresponding author; Dr A Suhrbier, QIMR Berghofer Medical Research Institute, Locked Bag 2000 Royal Brisbane Hospital, Qld. 4029, Australia; Tel: +61-7-33620415. Fax: +61-7-33620111. E-mail: Andreas.Suhrbier@qimrberghofer.edu.au

#Joint 1st WAS, TDH and TTL

J.E. currently at: The University of Queensland Diamantina Institute Translation Research Institute Princess Alexandra Hospital Woolloongabba, QLD 4102 Australia

D.P. currently at: the School of Natural Sciences and Queensland Micro- and Nanotechnology Centre, Griffith University, Nathan, Australia

a

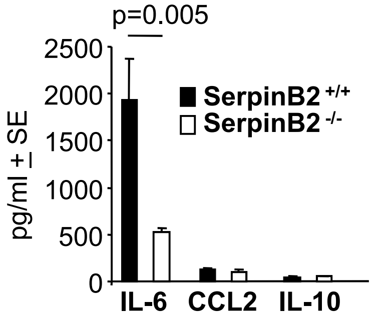

b

Rank USR	p-value	Act. z-score
1) NFKB1	4.36E-13	2.83
3) HMGB1	2.25E-12	3.09
4) RELA	3.80E-12	3.89
5) NFKBIA	7.8E-12	1.85
6) NR3C1	2.44E-11	-3.49
9) NFkB (complex)	2.01E-10	
20) RELB	3.08E-08	-0.257
34) NFKB2	7.15E-07	

c

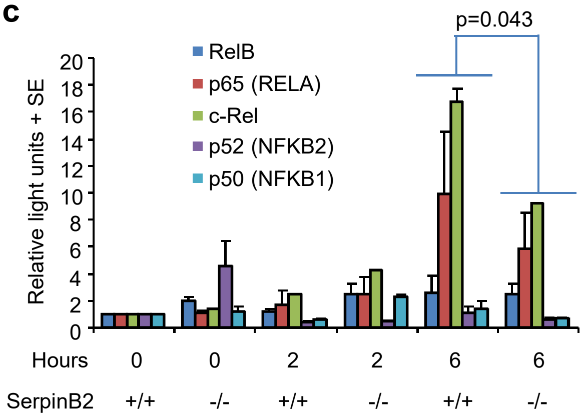

d

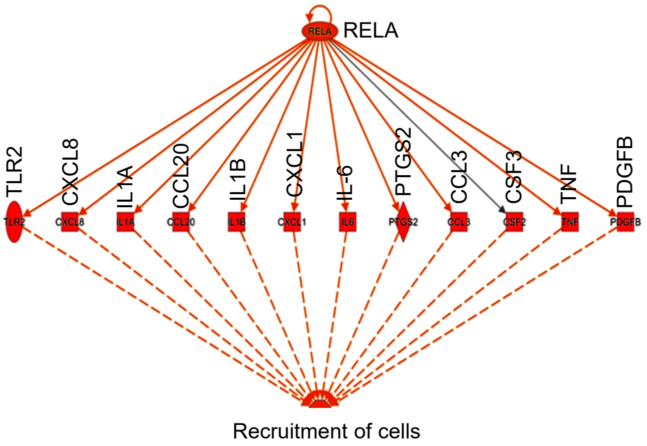

Fig. S1. CEMiTool validation and IPA analyses. (a) SerpinB2 expression increases IL-6 production**.** Basal cytokine/chemokine secretion of RPM from SerpinB2$^{-/-}$ and SerpinB2$^{+/+}$ mice cultured for 30 h on plastic. (n=6 mice per group, data from 2 independent experiments, statistics by Kolmogorov–Smirnov test). Cytokine levels determined by BD Cytokine Bead Array (BD Biosciences) as described (Schroder et al 2010). (**b**) Genes identified by CEMiTool (Table S1a) were analysed by IPA (117 genes were recognised by IPA) using direct only setting and giving a nominal expression value of 2 to co-regulated genes and -2 to counter regulated genes (wrt SerpinB2). Selected top USR identified by IPA are shown ranked by p value 1) being the most significant, and the position in the rank for each USR indicated. (**c**) In 2-3 independent experiments DNA binding of NF-κB family members was quantitated using nuclear extracts (as described Yekollu et al 2011 Diabetes 60; 2928-38) and the NFkB Family EZ-TFA Transcription Factor Assay Chemiluminescent Kit (Merck). SerpinB2$^{-/-}$ and SerpinB2$^{+/+}$ RPM were treated for the indicated time with anti-CD40 and IFNγ (as described Schroder *et al.*, 2010) . Statistics by related samples Wilcoxon Signed Rank test. (**d**) The *Top Regulator Effect Network* identified by IPA for the aforementioned 117 genes was "RELA Recruitment of cells"; consistency score 3.175.

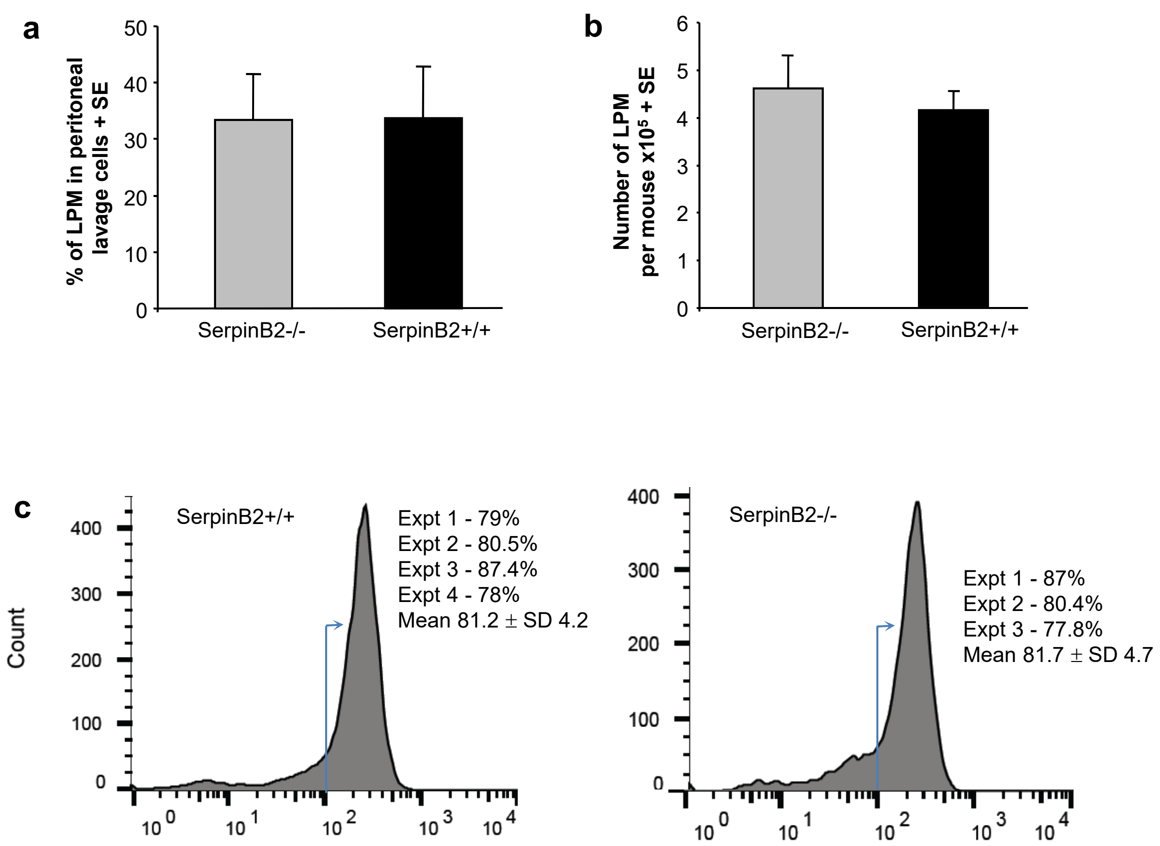

Fig. S2. LPM number and percentages. (**a**) Percentage of LPM in female SerpinB2$^{-/-}$ and SerpinB2$^{+/+}$ mice (data from 7 independent FACs experiments using pooled peritoneal lavage cells from 1-5 mice for each strain for each FACS run). p=0.7 (t test). (**b**) LPM numbers (F4/80^{hi}) per mouse from female SerpinB2$^{-/-}$ and SerpinB2$^{+/+}$ mice (data from 4 independent FACs analyses using pooled peritoneal lavage cells from 2-4 mice for each strain for each FACs run). p=0.59 (t test). (**c**) Peritoneal lavage cells were plated for 3 hours in 6 well plates, washed 5 times in PBS to remove non-adherent cells, cultured for 24 hours. For FACS analyses cells were scraped and stained with F4/80 antibody. Profile of cells after gating out dead cells (propidium iodide^{hi}) for Expt 1 is shown and percentages for repeat experiments added in the figure. Each experiment used 2 female SerpinB2$^{-/-}$ and SerpinB2$^{+/+}$ mice.

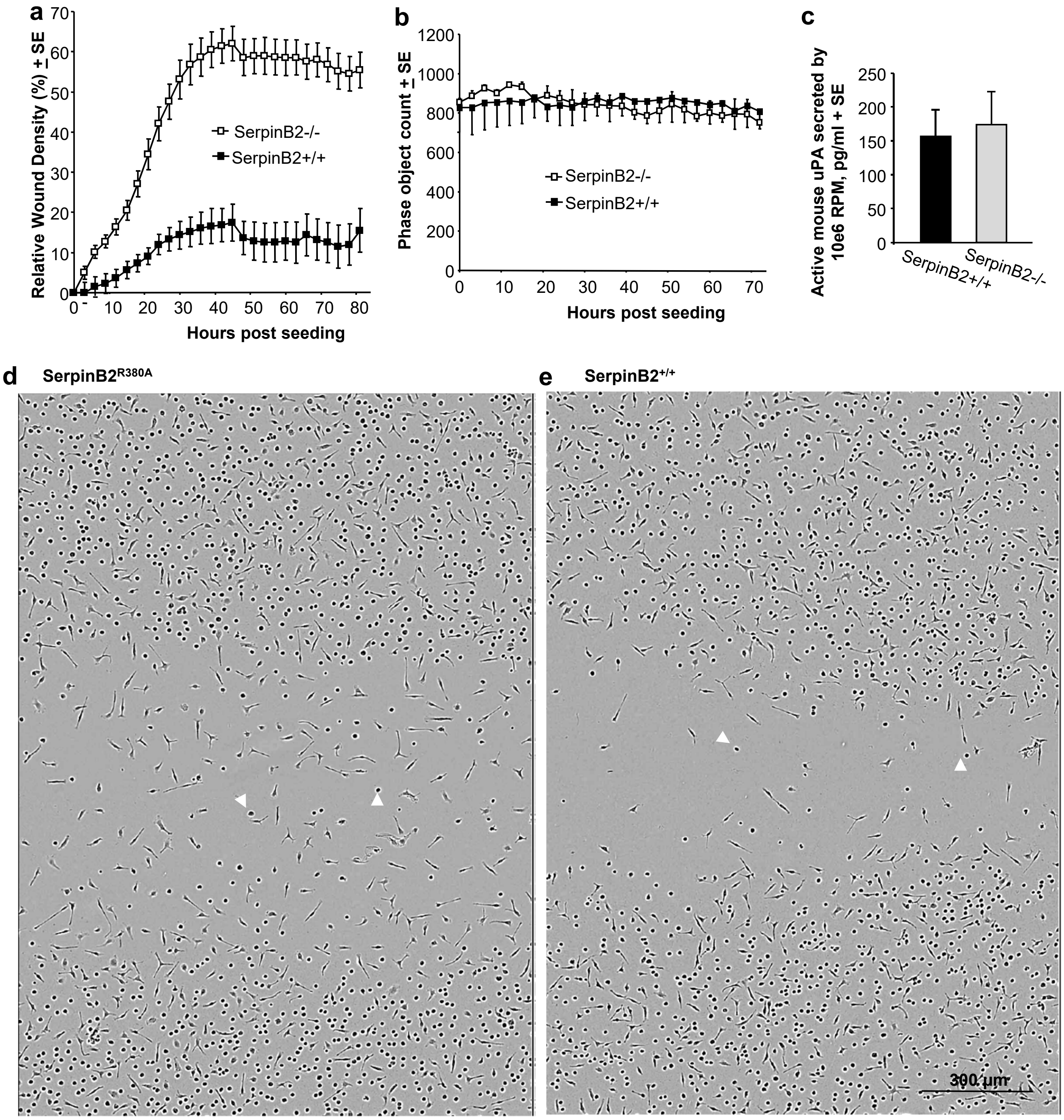

Fig. S3. Incucyte analyses of RPM from SerpinB2$^{-/-}$ and SerpinB2$^{+/+}$ mice and secretion of active uPA by RPM. **(a)** Repeat experiment to that shown in Fig. 3a , except using one pool for each mouse strain, each derived from 3 mice and 15-18 wells for each pool. SE was derived from the 15-18 wells. **(b)** Survival data for wells analysed in Fig. 3a for cells outside the wound/scratch area. **(c)** RPM from 10 mice per group were cultured overnight on plastic and supernatants assayed in duplicate for active uPA using the Mouse uPA ELISA Kit (Innovative Research). The data is derived from 2 independent experiments (n=10). (**d**) Phase image showing scratch wound at 27 hrs for SerpinB2^{R380A} RPM. (**e**) Phase image showing scratch wound at 27 hrs for SerpinB2$^{+/+}$ (C57BL/6J) RPM. Round cells in the scratch wound area (white arrowheads) were deemed non-migrating and were not included in the calculations for Figs. 3d and e. The number of round cells were not significantly different between SerpinB2+/+ and SerpinB2 deficient RPM in these experiments.

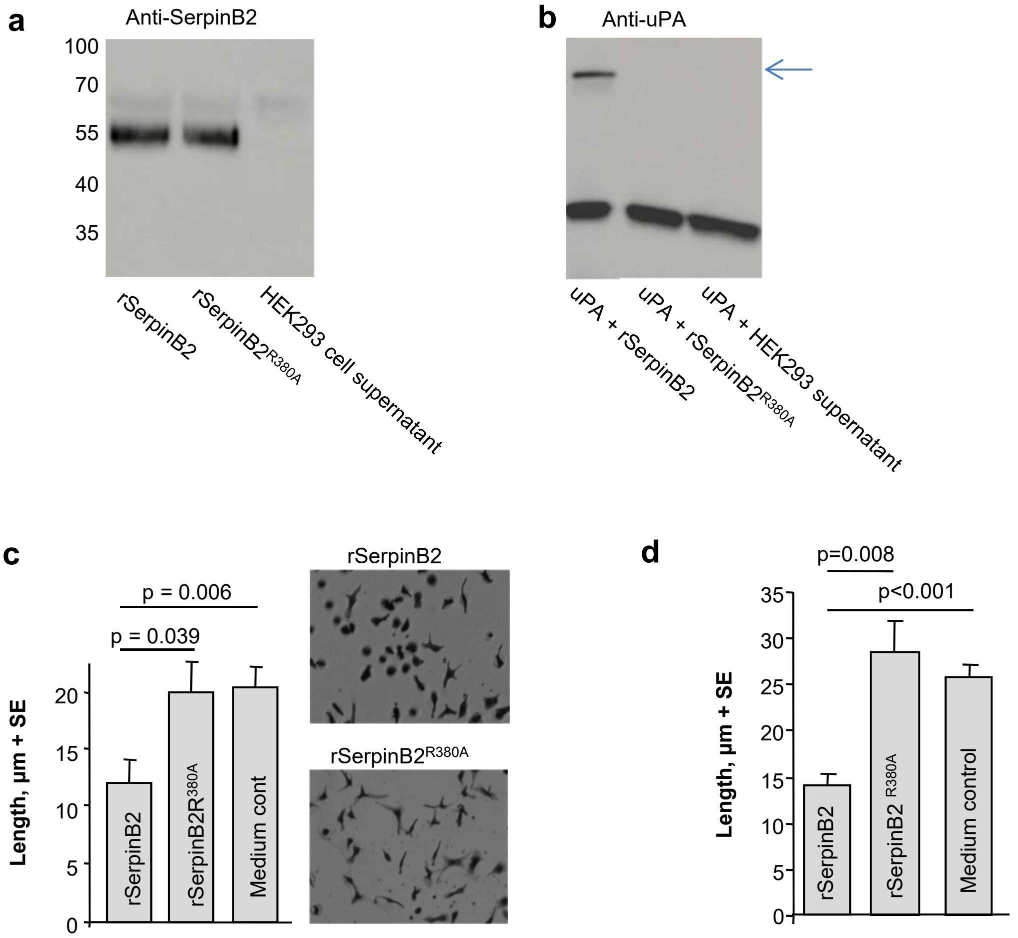

Fig. S4. Recombinant SerpinB2. (a) Recombinant murine SerpinB2 (rSerpinB2) and recombinant murine SerpinB2^{R380A} (rSerpinB2^{R380A}) were generated by transfecting HEK293T cells with plasmids (pLVX-IRES-ZsG) encoding rSerpinB2 or rSerpinB2^{R380A} both with a PAI-1 signal peptide. Supernatants were harvested, concentrated and analysed by immunoblotting using the anti-murine SerpinB2 antibody. Note higher molecular weight of glycosylated SerpinB2 proteins. (**b**) rSerpinB2 and rSerpinB2^{R380A} were incubated with murine uPA (Innovation Research, USA; 20 mins at 37°C) and analysed by immunoblotting using an anti-uPA antibody (Abcam, ab20789). Arrow indicates the covalent SerpinB2/uPA complex. As expected rSerpinB2^{R380A} did not form the complex. (**c**) RPM from SerpinB2$^{-/-}$ mice were seeded on Matrigel in the presence of 100 ng/ml LPS and supernatants containing ≈50 ng/ml rSerpinB2 or rSerpinB2^{R380A} for 24 hrs. Cells were fixed with paraformaldehyde, stained with crystal violet and the length of cellular protrusions measured as described (Schroder *et al* 2014). Statistics by t tests. (**d**) As for C except using B16 melanoma cells as described (Schroder *et al* 2014). Statistics by Kolmogorov-Smirnov test ($p=0.008$) and t test ($p<0.001$).

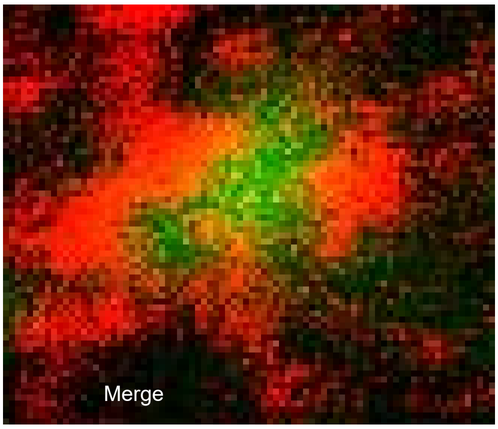

Color	Pixels	Percent
Green	14645	9.0
Red	67735	41.8
Yellow	8735	5.4

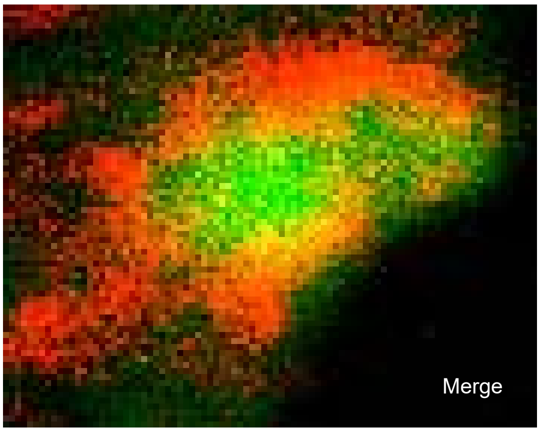

Color	Pixels	Percent
Green	37034	20.9
Red	65966	37.2
Yellow	26299	14.8

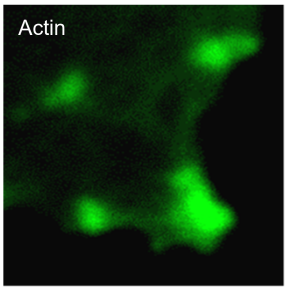

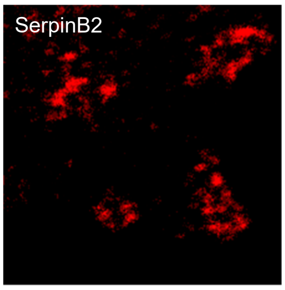

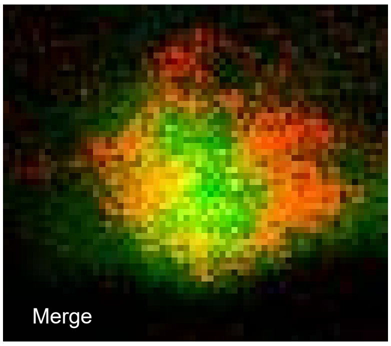

Color	Pixels	Percent
Green	19586	19.3
Red	23689	23.3
Yellow	13820	13.6

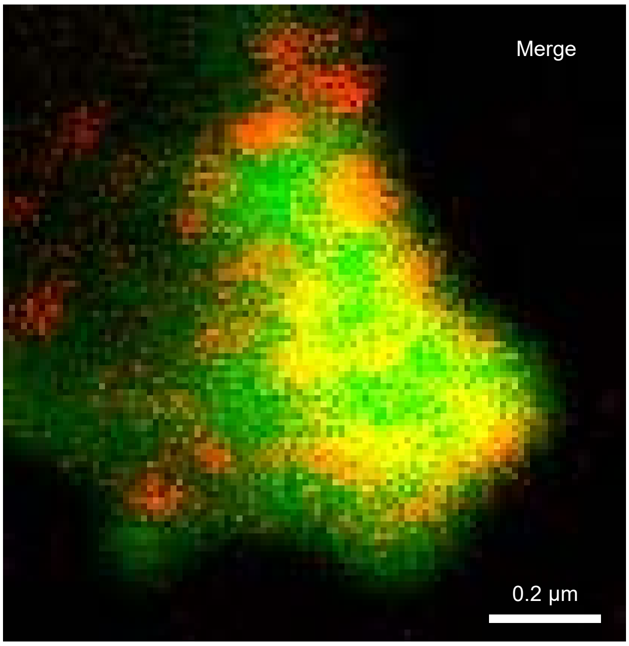

Color	Pixels	Percent
Green	63830	24.4
Red	53782	20.6
Yellow	41868	16.0

Fig. S5. Enlarged merged images of focal adhesions shown in Fig. 4a. The two central images are the same as those shown in Fig. 4a. The merged images for the four focal adhesion represent enlargements of the four focal adhesions shown in the merged imaged Fig. 4a (right hand image) . Green, red and yellow (merged) pixels quantitated using ImageJ Color Pixel Counter plugin. (Images represent 2-3 500 nm adjacent Z stacks although yellow staining was similarly seen in a single Z stack).

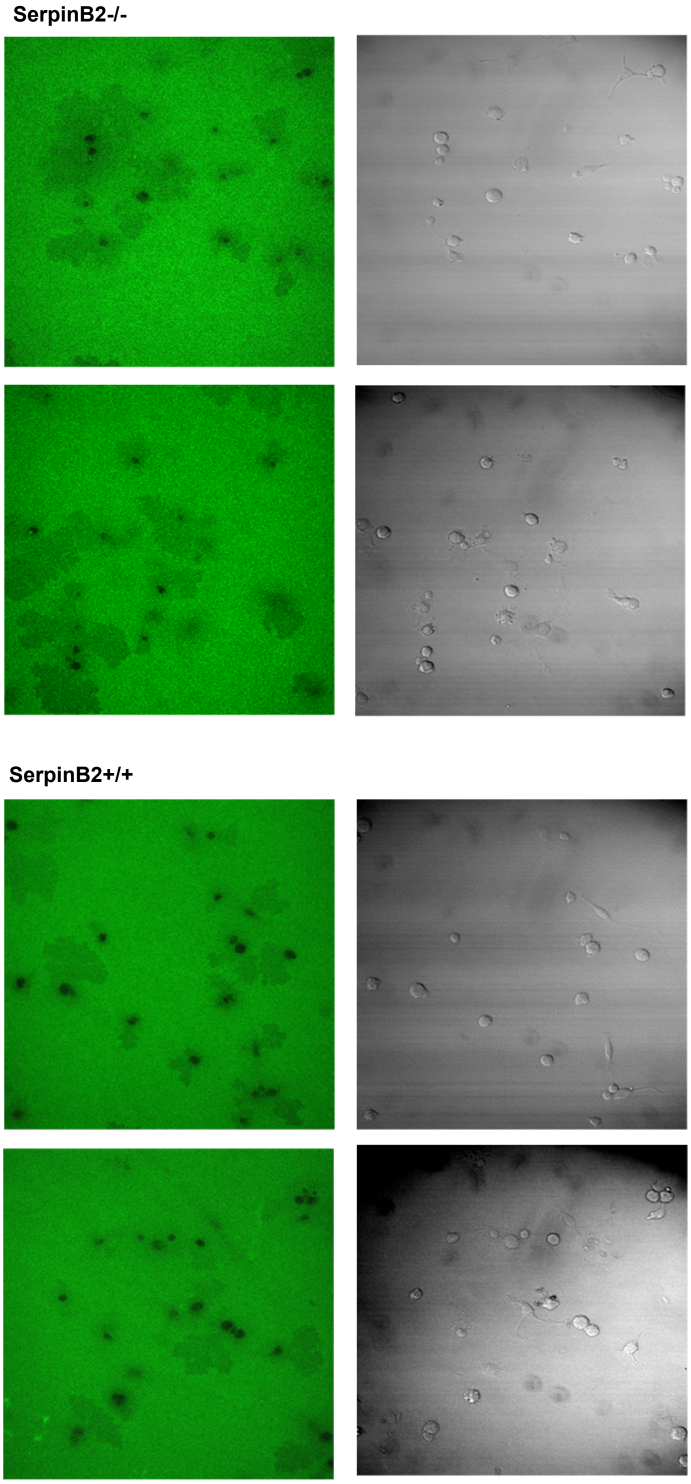

Fig. S6. RPM incubated on FITC gelatin. RPM from SerpinB2-/- and +/+ mice were incubated on FITC-gelatin for 24 hours as described (Fleetwood *et al* 2014). Black areas in the green "lawn" indicate peri-cellular proteolysis and loss of FITC, as viewed by confocal microscopy.

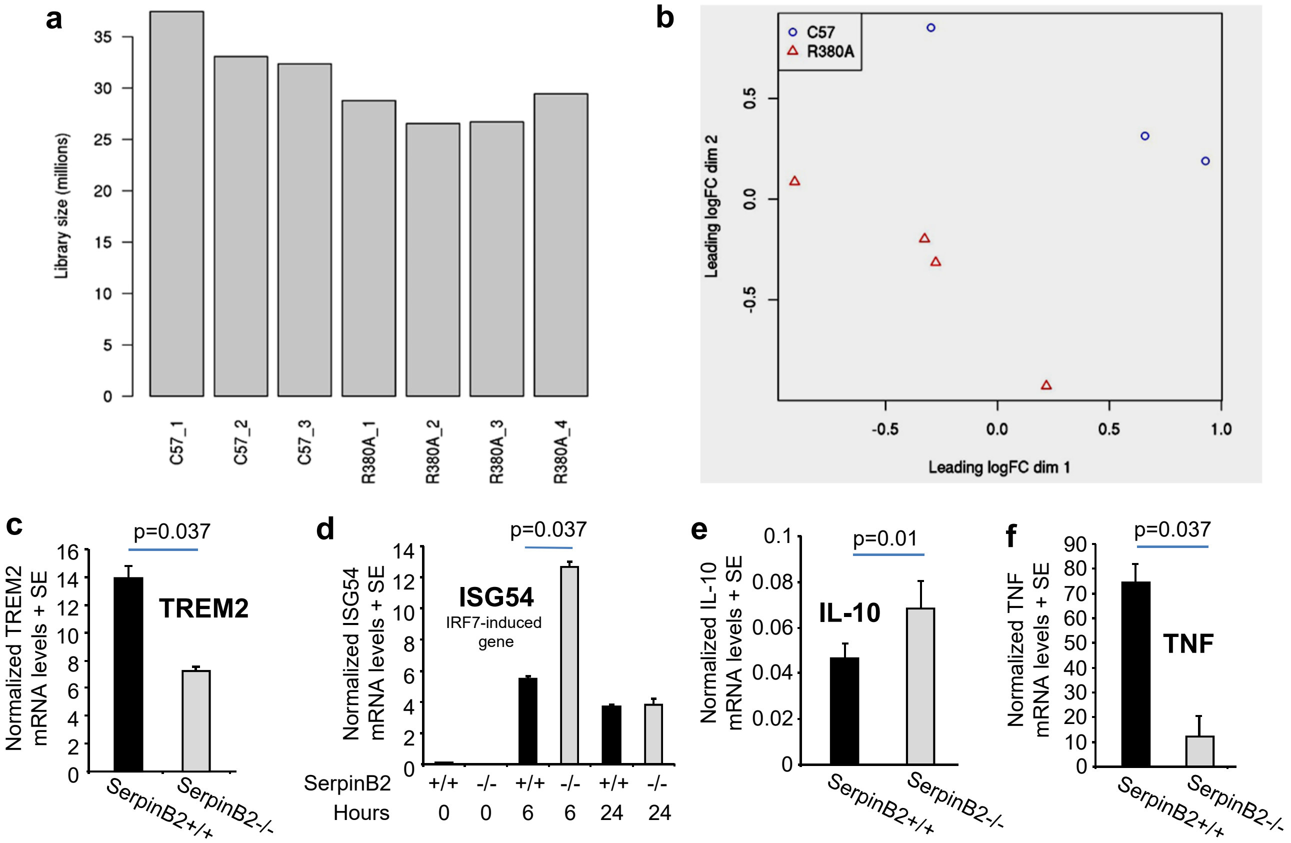

Fig. S7. **RNA-Seq QC data and validation.** (**a**) Normalized library sizes. (**b**) Normalized MDS plot of all samples. (**c**) RNA-Seq data showed 1.44 fold lower TREM2 mRNA in RPM from mice with inactive SerpinB2 (FDR=9.47x10E-11) (Table S1d). qRT PCR data normalized to RPL13A (Schroder *et al.* 2010) showed significantly lower TREM2 mRNA in SerpinB2$^{-/-}$ RPM (Statistics by Kolmogorov-Smirnov test, n=4). (**d**) RNA-Seq data showed 1.55 fold higher IRF7 mRNA in mice with inactive SerpinB2 (FDR=1.89x10E-5) (Table S1d). See also Table S1e where IRF7 is the top USR by z score. Here is shown qRT PCR data normalization to RPL13A for ISG54 mRNA induction (as described[1]) in RPM treated with polyinosinic:polycytidylic acid (polyIC) as described (Schroder *et al.* 2010). (Statistics by Kolmogorov-Smirnov test, n=4). Elevated constitutive IRF7 protein expression is associated with increased early type I interferon responses [2], which includes direct promotion of ISG54 transcription [3,4]. PolyIC treatment was used to activate IRF7 [5], with the increased levels of ISG54 mRNA at 6 hours post treatment consistent with constitutively elevated levels of IRF7 protein in SerpinB2$^{-/-}$ RPM. At 24 hours IRF7 induction [6] and polyIC degradation results in similar and reduced ISG54 induction. (**e**) RNA-Seq data showed 1.69 fold higher IL-10 mRNA in RPM from mice with inactive SerpinB2 (FDR=0.0051) (Table S1d). qRT PCR data normalized to RPL13A (Schroder *et al.* 2010) showed significantly higher IL-10 mRNA in SerpinB2$^{-/-}$ RPM (Statistics by Kolmogorov-Smirnov test, n=12, 3 experiments with 4 replicates each). (**f**) RNA-Seq data showed 1.45 fold lower TNF mRNA in RPM from mice with inactive SerpinB2 (FDR=4.16x10E-7) (Table S1d). qRT PCR data normalized to RPL13A (Schroder *et al.* 2010) showed significantly lower TNF mRNA in SerpinB2$^{-/-}$ RPM (Statistics by Kolmogorov-Smirnov test, n=4).

1 Poo, Y. S. *et al.* Multiple immune factors are involved in controlling acute and chronic CHIKV infection. *PLoS Negl Trop Dis* **8**, e3354 (2014).
2 Webster, B. *et al.* Plasmacytoid dendritic cells control dengue and chikungunya virus infections via IRF7-regulated interferon responses. *Elife* **7** e34273 (2018).
3 Daffis, S. *et al.* Interferon response factor 7, IRF-7 induces the antiviral alpha interferon response and protects against lethal West Nile virus infection. *J Virol* **82**, 8465-8475 (2008).
4 Reich, N. C. A death-promoting role for ISG54/IFIT2. *J Interferon Cytokine Res* **33**, 199-205 (2013).
5 Prow, N. A. *et al.* Lower temperatures reduce type I interferon activity and promote alphaviral arthritis. *PLoS Pathog* **13**, e1006788 (2017).
6 Rudd, P. A. *et al.* Interferon response factors 3 and 7 protect against Chikungunya virus hemorrhagic fever and shock. *J Virol* **86**, 9888-9898 (2012)

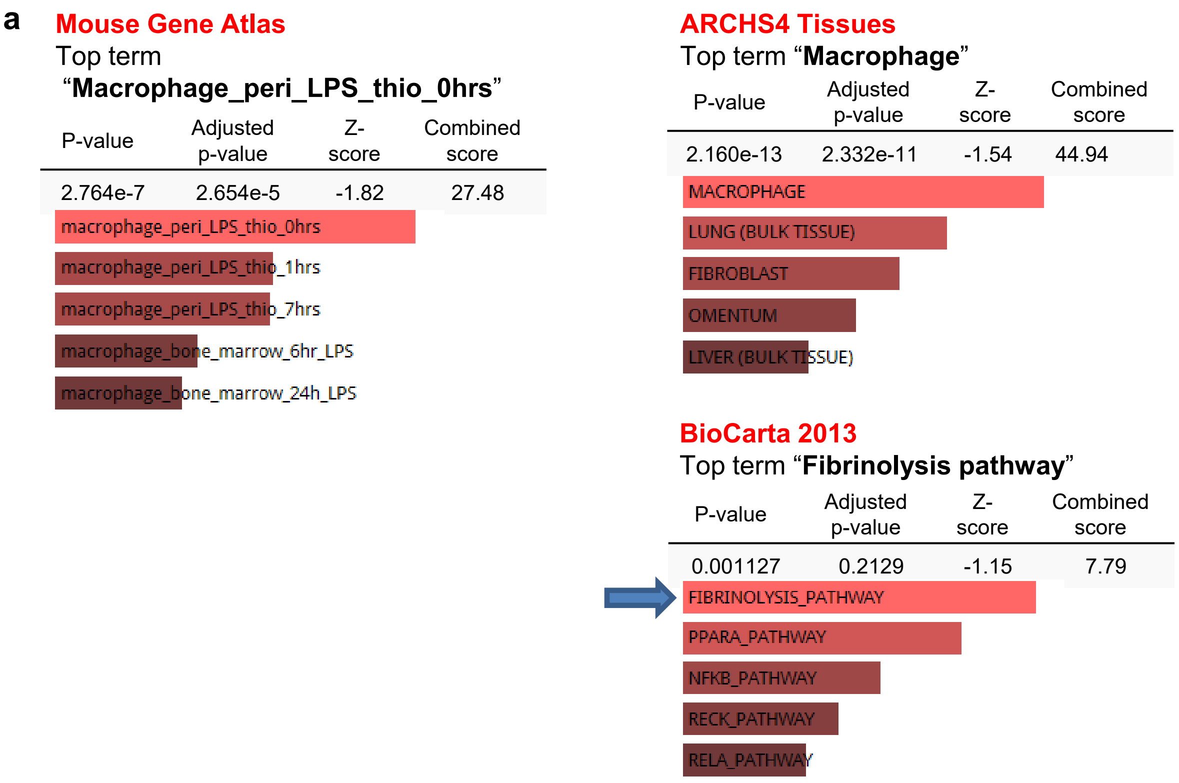

b **Molecular and Cellular Functions**

Name	p-value	#Molecules
Cellular Movement	1.46E-12 - 6.56E-59	465
Cell Death and Survival	6.16E-13 - 6.14E-39	557
Cellular Function and Maintenance	1.60E-12 - 5.67E-37	574
Cell-To-Cell Signaling and Interaction	2.17E-12 - 1.01E-34	376
Cellular Assembly and Organization	1.14E-12 - 2.01E-31	368

Physiological System Development and Function

Name	p-value	#Molecules
Immune Cell Trafficking	2.17E-12 - 8.81E-53	297
Tissue Morphology	3.27E-13 - 8.96E-53	430
Hematological System Development and Function	2.17E-12 - 5.26E-46	438
Organismal Survival	6.03E-28 - 2.40E-41	463
Lymphoid Tissue Structure and Development	3.27E-13 - 1.13E-34	319

Fig. S8. Bioinformatics analyses. (**a**) All DEGs (1481 genes, q<0.01) from RNA-Seq analysis of RPM from SerpinB2^{R380A} and C57BL/6 mice migrating on Matrigel were analysed using Enrichr http://amp.pharm.mssm.edu/Enrichr/). Bar charts are plotted according to the Combined score ranking; p values and scores are provided for the top scoring term (top bar) . Blue arrow; Fibrinolysis pathway. (**b**) IPA (direct only) of the DEG list (1481 genes, q<0.01) illustrating that the most significant *Molecular and Cellular Function* was *Cellular Movement* and the most significant *Physiological System Development and Function* was *Immune Cell Trafficking*.

BioCarta 2016
Rho cell motility signaling pathway

P-value	Adjusted p-value	Z-score	Combined score
3.404e-4	0.05446	-1.47	11.76

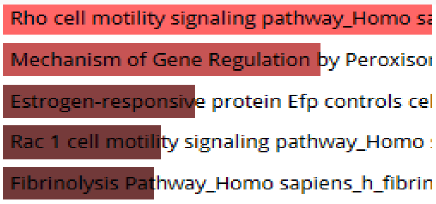

KEGG 2015
Focal adhesion

P-value	Adjusted p-value	Z-score	Combined score
2.084e-7	0.00002418	-2.18	33.51

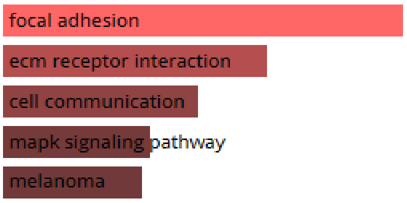

WikiPathways 2015
Focal adhesion

P-value	Adjusted p-value	Z-score	Combined score
5.016e-6	0.001500	-2.14	26.06

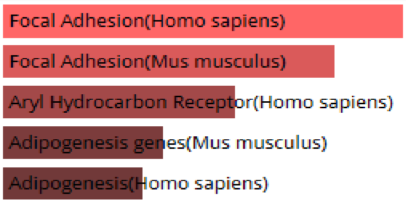

GO Biological Process 2013
Regulation of cell-matrix adhesion

P-value	Adjusted p-value	Z-score	Combined score
8.058e-6	0.004130	-2.18	25.57

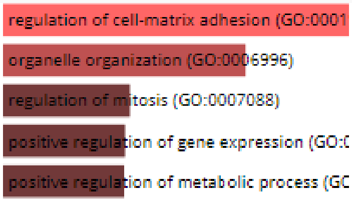

Reactome 2016
Extracellular matrix organization

P-value	Adjusted p-value	Z-score	Combined score
6.142e-7	0.0001087	-1.69	24.12

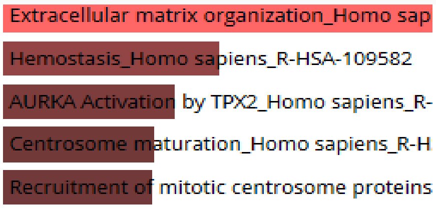

NCI-Nature 2016
Beta1 integrin cell surface interactions

P-value	Adjusted p-value	Z-score	Combined score
6.142e-7	0.0001087	-1.69	24.12

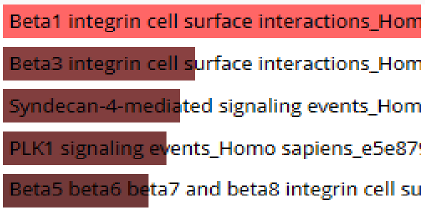

Go Molecular Function 2018
Actin binding

P-value	Adjusted p-value	Z-score	Combined score
6.142e-7	0.0001087	-1.69	24.12

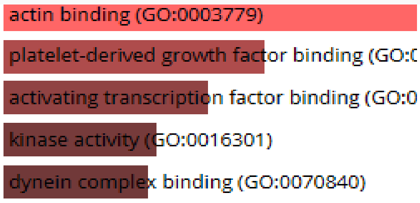

Panther 2016
Integrin signalling pathway

P-value	Adjusted p-value	Z-score	Combined score
0.002926	0.07913	-1.78	10.39

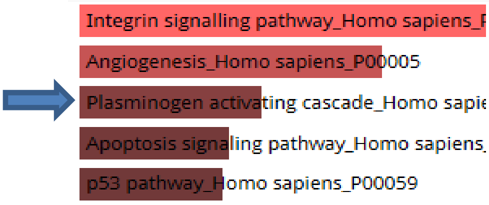

Fig. S9. The down-regulated DEGs (857 genes, q<0.01) in RPM from SerpinB2^{R380A} mice were analysed using Enrichr (http://amp.pharm.mssm.edu/Enrichr/). Bar charts are plotted according to the Combined score ranking; p values and scores are provided for the top scoring terms (top bar) . Blue arrow indicates Plasminogen activating cascade p=0.00313, q=0.07913, Z score = -1.09, Combined score=6.27.

a

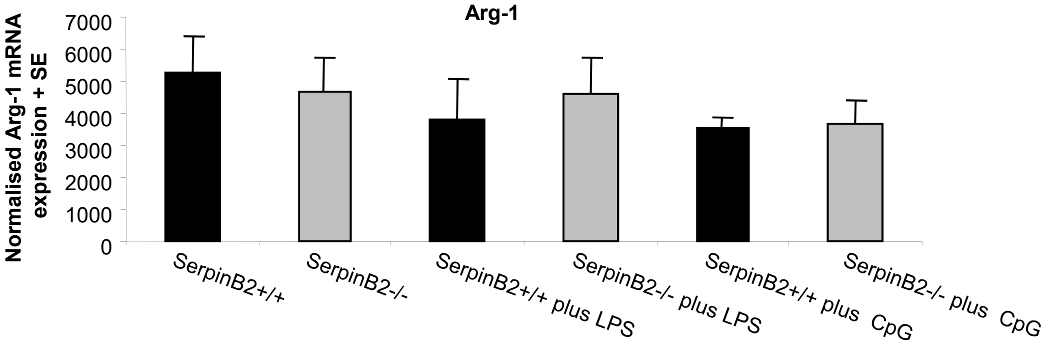

Fig. S10a. Arg-1 mRNA levels in SerpinB2$^{-/-}$ and SerpinB2$^{+/+}$ RPM. RPM were left untreated or were treated with 100 ng/ml LPS or 2.5 µM CpG, and qRT PCR performed and normalised to RPL13A (as described Schroder et al 2010). Arg-1 mRNA levels at 6 hours post treatment are shown (see Schroder et al 2014 for primers). Mean of data from 2-3 independent experiments performed in duplicate using pooled RPM from 3-5 mice.

b

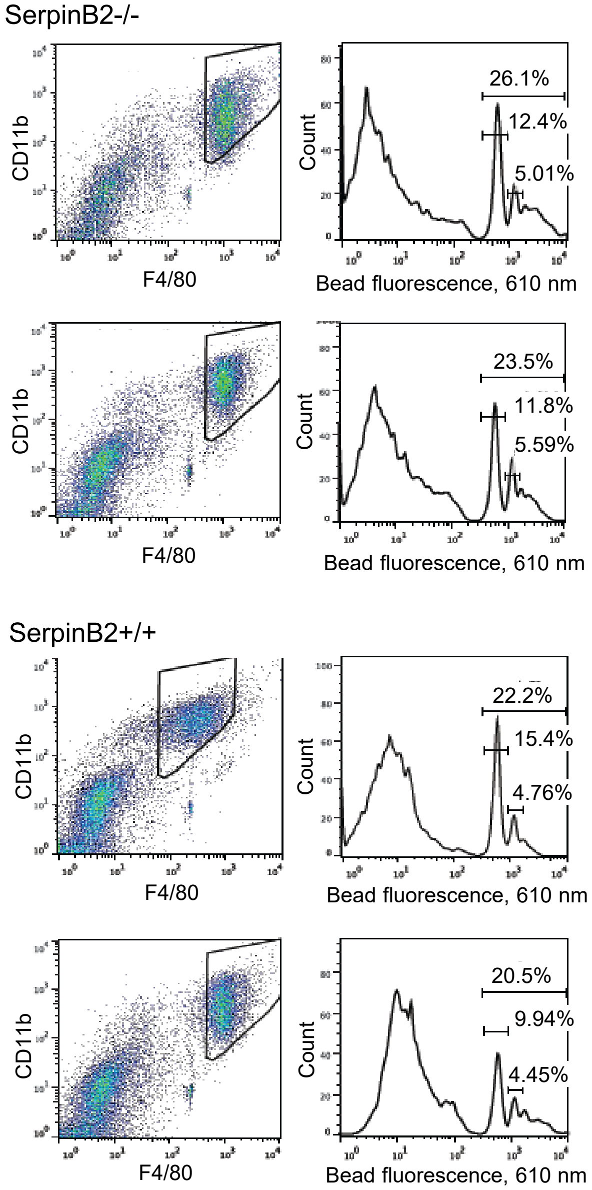

Fig. S10b. *In vivo* phagocytosis of fluorescent beads by LPM. Mice (n=2 for each strain) were injected i.p. with 2 µm carboxylate-modified polystyrene red fluorescent beads (Sigma, L3030) and after 45 min peritoneal cells were collected by lavage. Cells were analysed by FACs as in Fig. 2a with LPM gated (CD11b^{hi} and F4/80^{hi} cells in the dot blots). Gated cells were then analysed on a third channel (610 nm) to measure bead fluorescence (histogram plots).

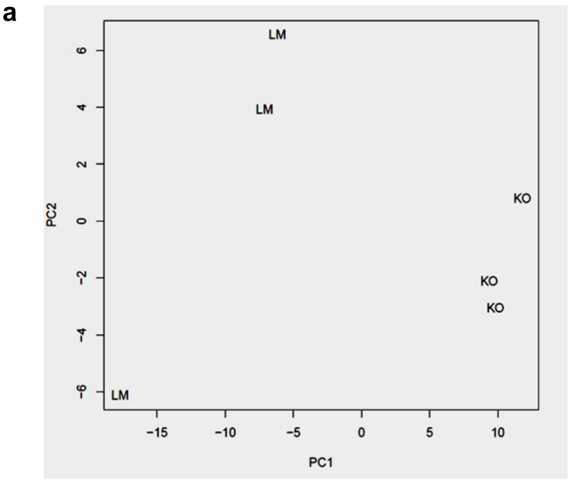

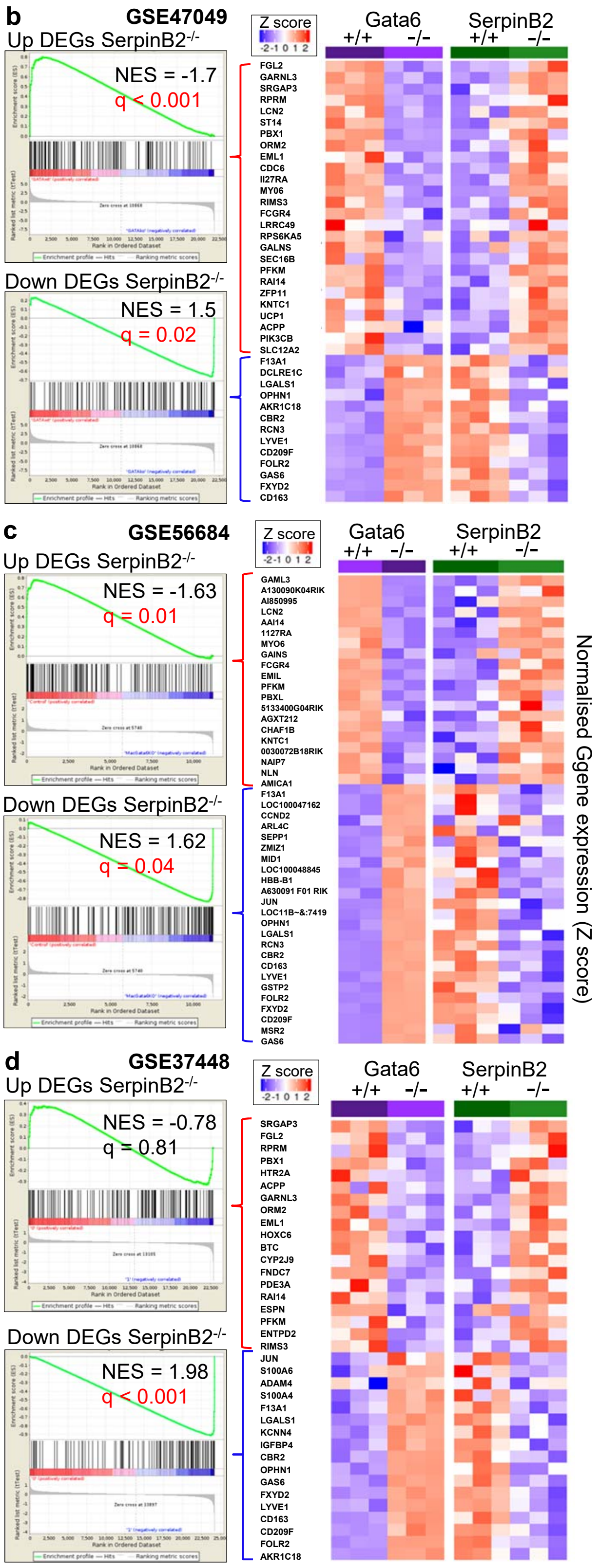

Fig. S11. SerpinB2$^{-/-}$ vs SerpinB2$^{+/+}$ LPM microarray and GSEA. Peritoneal cells were obtained by lavage from naïve SerpinB2$^{-/-}$ and SerpinB2$^{+/+}$ mice. F4/80^{hi} cells (Fig. 2a) were isolated by FACS sorting from 3 separate sorts per mouse strain using cells from 4 mice per sort to produce 6 samples (3 for SerpinB2$^{-/-}$ and 3 for SerpinB2$^{+/+}$ mice). These cells were >90% F4/80^{hi} CD11b^{hi} LPM (Fig. 2a). Biotinylated cRNA was prepared with the Illumina TotalPrep RNA Amplification Kit (Ambion/Applied BioSystems, Austin, TX, USA). Labelled cRNA was hybridized to MouseWG-6 v2.0 BeadChip Arrays (Illumina Inc, San Diego, CA, USA), and then washed and scanned using the iScan (Illumina) according to standard protocols. Data were extracted in GenomeStudio (Illumina) using default settings and further processed in R [1] using the Lima package. Arrays were background corrected using negative controls followed by quantile normalization using negative and positive controls. Probes that had a detection p-value >0.05 in more than 3 samples were considered non-expressed and discarded. Differentially expressed probes were identified by fitting linear models to the expression data of each gene and using a moderated t-statistic test [2] after applying estimated quality weights to each array [3]. A list of differentially expressed genes (p<0.05) was generated (Table S1i).

1. R Core Team, www.R-project.org, Vienna, Austria (2018)
2. Smyth, G. K.. *Stat Appl Genet Mol Biol* **3**, Article3 (2004).
3. Ritchie, M. E. *et al. BMC Bioinformatics* **7**, 261 (2006).

(**a**) Microarray PCA plot for the 3 replicate LPM samples for SerpinB2$^{-/-}$ (KO) and SerpinB2$^{+/+}$ (LM) mice using differentially expressed genes. (**b-d**) GSEAs as in Fig. 6 but using the DEGs from the microarray analysis.

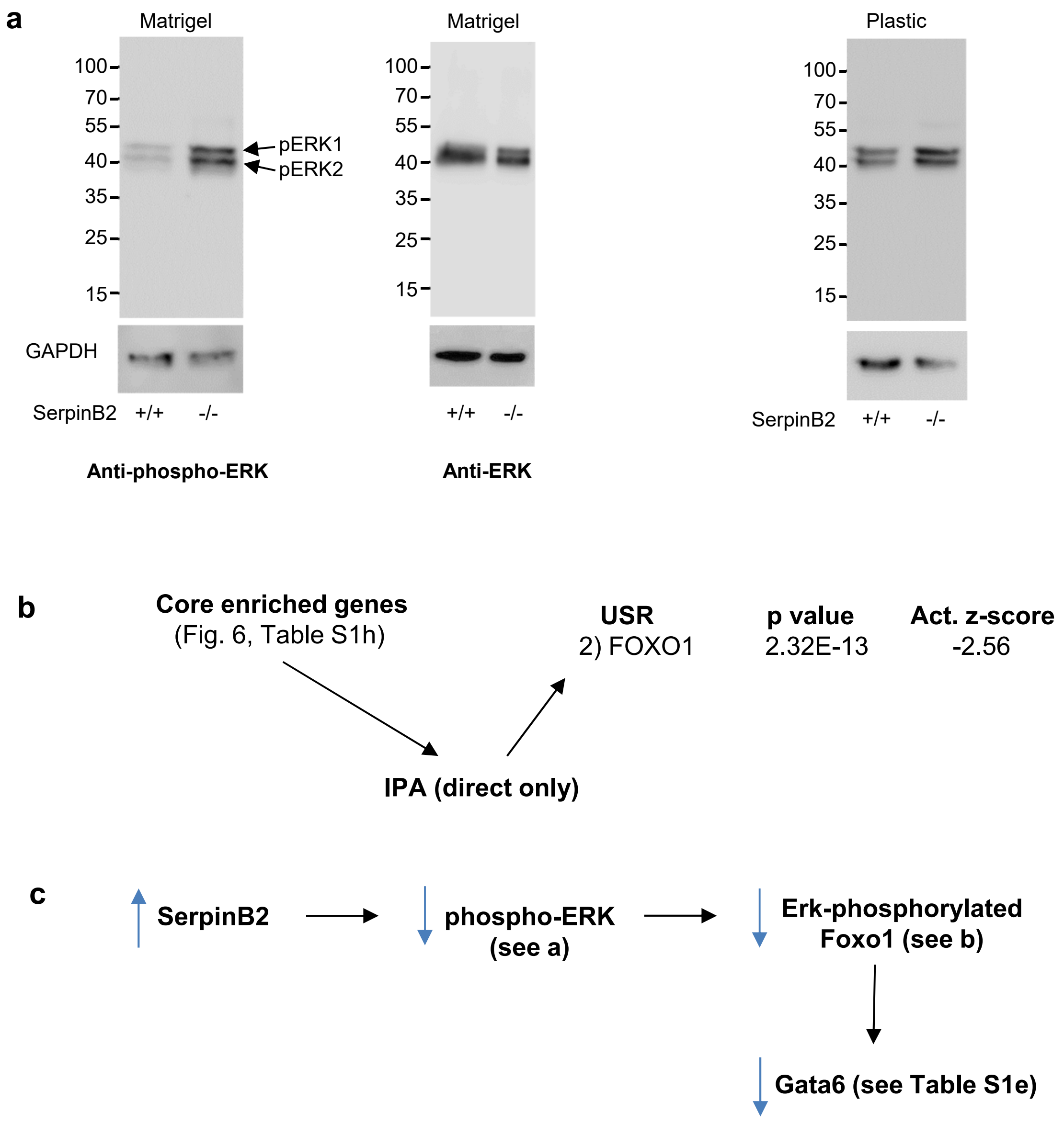

Fig. S12. Phospho-ERK1/2 immunoblotting and potential role in the counter regulation of Foxo1 and Gata6. (a) RPM from SerpinB2$^{-/-}$ and SerpinB2$^{+/+}$ mice were cultured for 37 hrs on Matrigel or plastic and were then analysed by Western blotting using an anti-phospho-MAPK family (Erk1 & Erk2) monoclonal antibody (L34F12; Cell Signalling Technology). The same samples were run on a separate gel and analyzed using anti-ERK antibody (137F5 Rabbit mAB #4695; Cell Signalling Technology. **(b)** The core enriched genes from Fig. 6 (listed in Table S1h with genes up-regulated in SerpinB2+/+ RPM given a nominal fold change of +2 and those down-regulated given a nominal fold change of -2) were analyzed by IPA (using the direct only option). The second most significant USR was FOXO1 with a negative activation z-score, suggesting SerpinB2 expression is associated with reduced FOXO1 activity. **(c)** A speculative mechanism whereby SerpinB2 expression might counter-regulate Gata6 activity. Note: Akt-mediated phosphorylation of FOXO1 is distinct and inhibits FOXO1 activity (Cabrera-Ortega et al 2017).

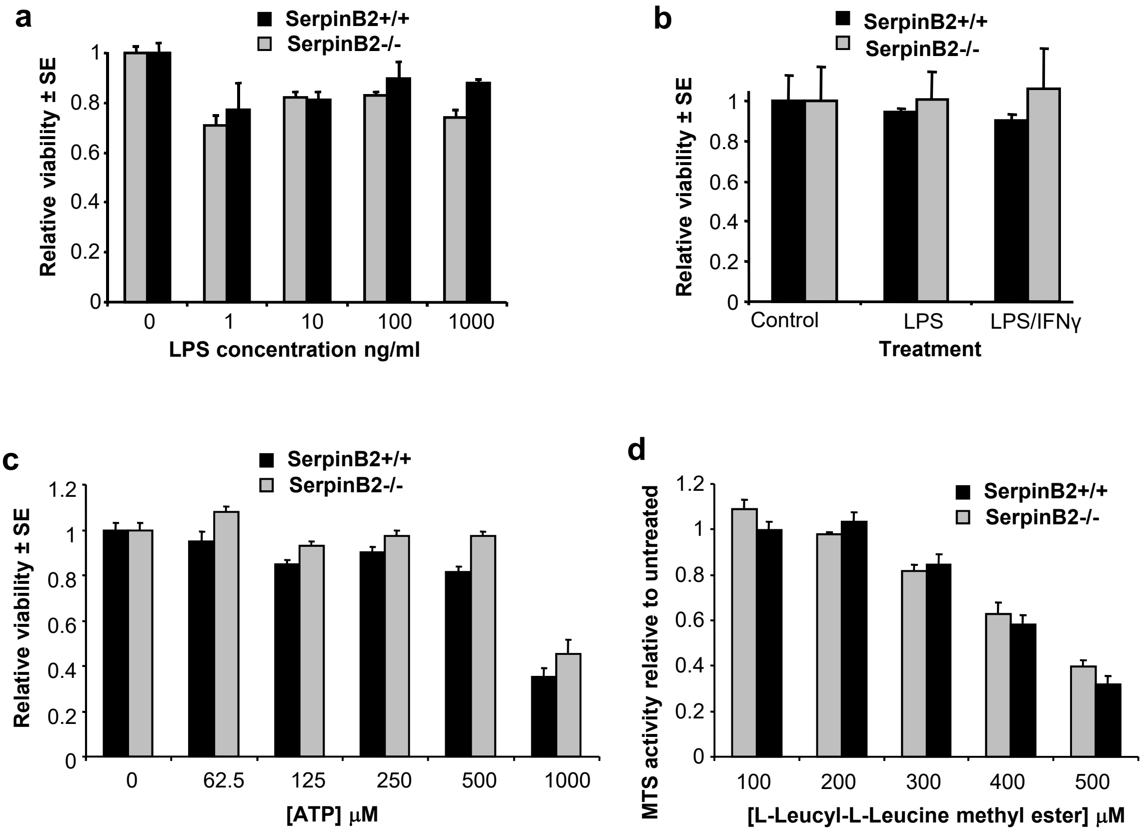

Fig. S13. RPM survival after LPS, IFNγ, ATP and Leu-Leu-OMe treatments. (a) RPM were isolated by adherence (Schroder et al 2010) and cultured overnight, the indicated concentration of LPS was then added for 24 h, and cell viability was determined by crystal violet protein staining. Similar data was obtained at 48 h (data not shown). **(b)** RPM were isolated by adherence and treated with LPS (100 ng/ml) and IFNγ (20 IU) overnight and cell viability determined by crystal violet protein staining. **(c)** RPM were isolated by adherence and treated with LPS (100 ng/ml) and indicated concentrations of ATP overnight and cell viability determined by crystal violet protein staining. **(d)** RPM were isolated by adherence and treated overnight with L-Leucyl-L-Leucine methyl ester, a lysosomoptropic agent that induces caspase-independent cell death. Mycobacterium induces caspase-independent cell death in macrophages (O'Sullivan et al 2007 Infect Immun 75;1984-93) and SerpinB2 was reported to modulate mycobacterium induced cell death in macrophages (Gan et al 2008 Nat Immunol 9;1189-97). Cell viability was determined by MTS assay.

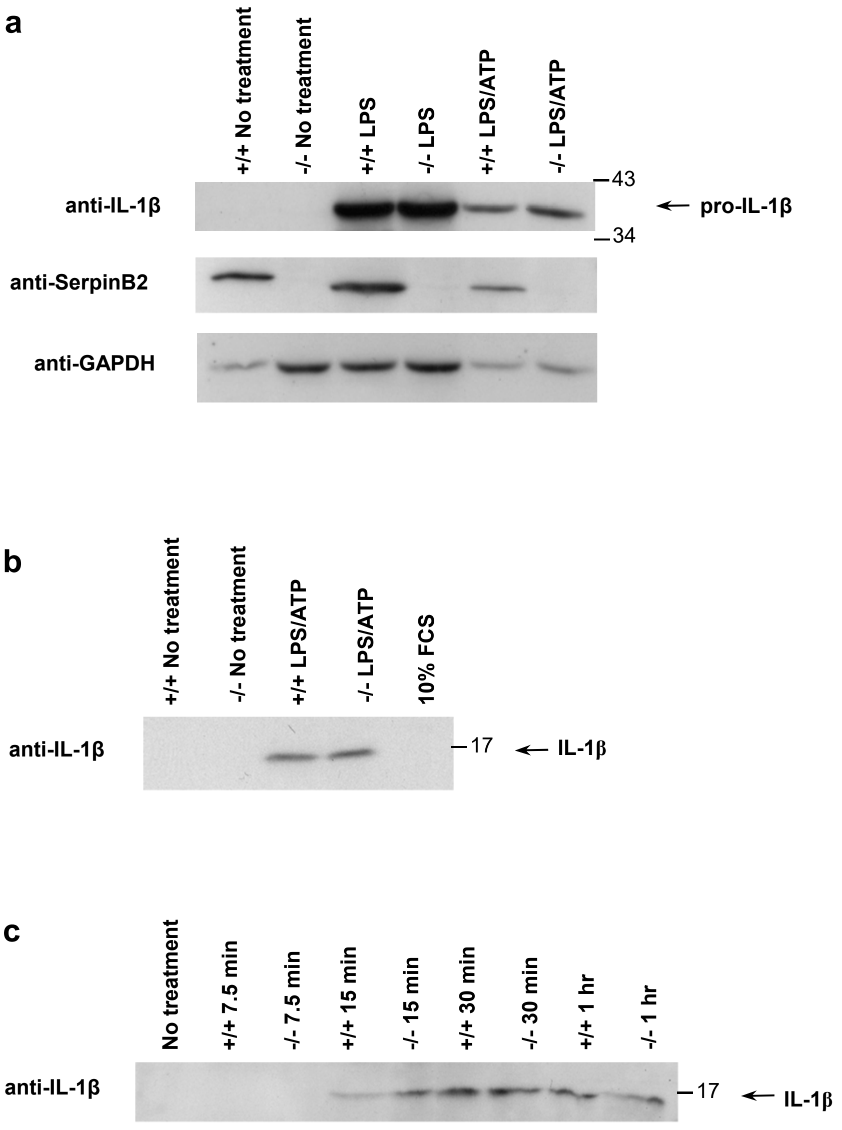

Fig. S14. IL-1β induction and secretion. (a) Pro-IL-1β protein induction was not significantly altered in SerpinB2$^{-/-}$ RPM. RPM were treated with 100 ng/ml LPS for 2 h and 1 mM ATP for 30 min and whole cell lysates were then analysed by Western; IL-1β antibody, IL-IF2 goat (R&D Systems) with donkey anti-goat secondary (Santa Cruz Biotechnology, Dallas, TX). **(b & c)** IL-1β secretion was not significantly altered in SerpinB2$^{-/-}$ RPM. RPM from SerpinB2$^{-/-}$ and $^{+/+}$ mice were treated with 100 ng/ml LPS and 1 mM ATP for 24 hours (b), or with LPS for 2 hours and for the indicated time with 1 mM ATP (c). Supernatants were immunoblotted with IL-1β antibody.

Anti-GAPDH

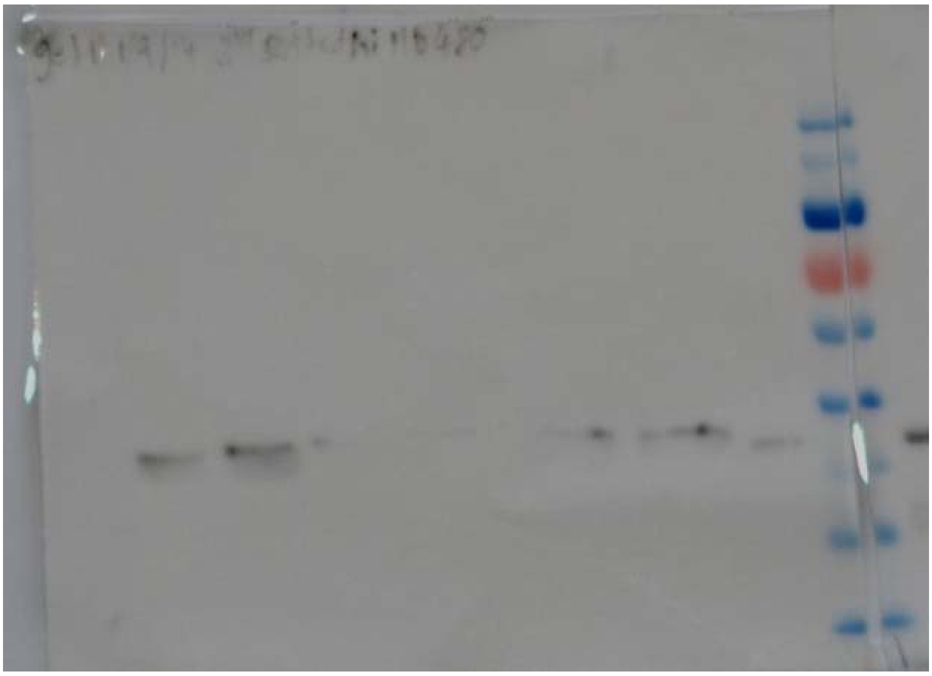

-/- +/+
SerpinB2 mice

Anti-murine SerpinB2 antibody

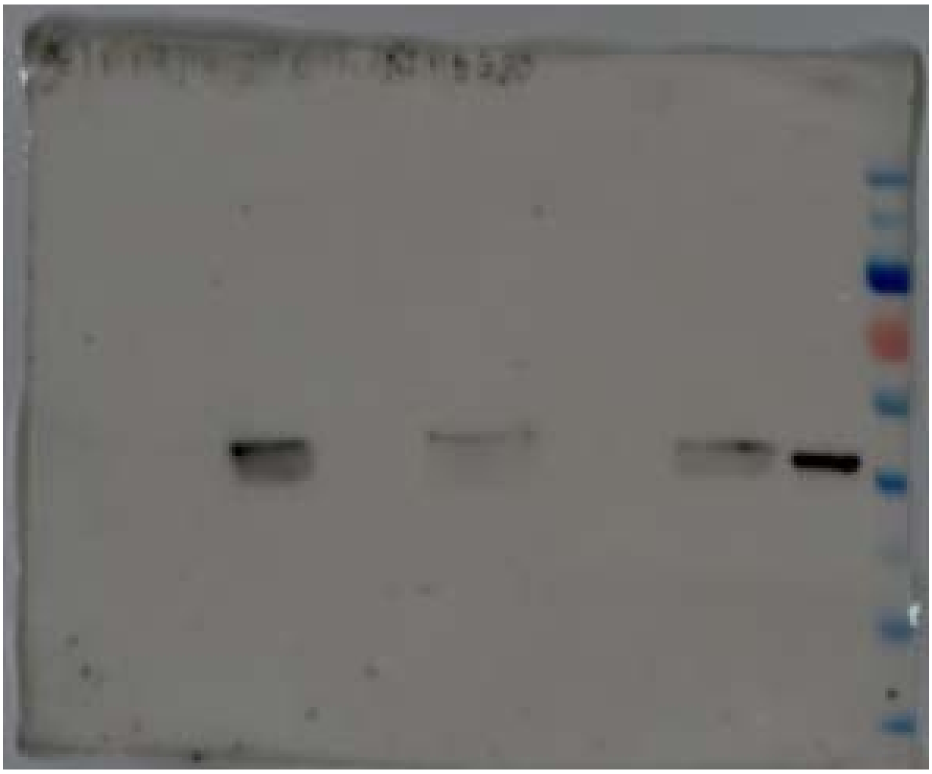

-/- +/+
SerpinB2 mice

Fig. S15. Full length blots for Figure 2C.
